# Establishment of a humanized patient‐derived xenograft mouse model of high‐grade serous ovarian cancer for preclinical evaluation of combination immunotherapy

**DOI:** 10.1002/1878-0261.70231

**Published:** 2026-03-14

**Authors:** Luka Tandaric, Line Bjørge, Martine Rott Lode, Cecilie Fredvik Torkildsen, Pia Aehnlich, Rammah Elnour, Daniela Elena Costea, Lars Andreas Akslen, Liv Cecilie Vestrheim Thomsen, Emmet McCormack, Katrin Kleinmanns

**Affiliations:** ^1^ Centre for Cancer Biomarkers CCBIO, Department of Clinical Science University of Bergen Norway; ^2^ Department of Obstetrics and Gynecology Haukeland University Hospital Bergen Norway; ^3^ Precision Oncology Research Group, Department of Clinical Science University of Bergen Norway; ^4^ Kinn Therapeutics AS Bergen Norway; ^5^ Department of Obstetrics and Gynecology Stavanger University Hospital Stavanger Norway; ^6^ Department of Obstetrics and Gynecology Heidelberg University Hospital Germany; ^7^ Department of Clinical Medicine Centre for Cancer Biomarkers CCBIO and Gade Laboratory of Pathology University of Bergen Norway; ^8^ Department of Pathology Laboratory Clinic, Haukeland University Hospital Bergen Norway; ^9^ Department of Clinical Medicine Centre for Cancer Biomarkers CCBIO Section for Pathology, University of Bergen Norway; ^10^ Department of Pathology Haukeland University Hospital Bergen Norway; ^11^ Department of Health Registry Research and Development Norwegian Institute of Public Health Oslo Norway; ^12^ Centre for Pharmacy, Department of Clinical Science University of Bergen Norway; ^13^ Department of Internal Medicine, Hematology Section Haukeland University Hospital Bergen Norway

**Keywords:** humanized mice, immuno‐oncology, immunotherapy, mouse model, ovarian cancer, patient‐derived xenograft

## Abstract

The limited efficacy of immunotherapy in clinical trials in high‐grade serous ovarian cancer (HGSOC) may improve by implementing experimental models that are more reflective of human biology into preclinical studies. To address this, we developed and validated a humanized patient‐derived xenograft mouse model of HGSOC. Human hematopoietic stem cells and patient‐derived HGSOC cells were engrafted into immunodeficient mice. The mice were administered durvalumab (anti‐PD‐L1) and/or oleclumab (anti‐CD73) immunotherapy intraperitoneally twice a week for 5 weeks. The treatment showed good tolerability with no observed side effects, though it failed to elicit a measurable antitumor response. Leukocytes in primary tumors were analyzed immunohistochemically, and circulating T cells were characterized using spectral flow cytometry. All tumors exhibited an immune‐excluded immunophenotype. No significant inter‐group differences in disease burden, intratumoral leukocyte density, or circulating T cells were observed. In the durvalumab‐only group, tumor burden significantly positively correlated with intratumoral cytotoxic and regulatory T‐cell densities. This model reflects the immunotherapy resistance of human disease in line with clinical findings, providing a robust platform for studying tumor–immune interactions and immunosuppressive mechanisms in HGSOC.

**Impact statement**

Our results address the critical need for representative preclinical models for testing combination immunotherapy in HGSOC by providing a robust preclinical platform that can enhance the reliability of preclinical data and contribute to the improvement of the design and outcomes of future clinical trials.

## Graphical abstract

We have established a humanized orthotopic patient‐derived xenograft (Hu‐oPDX) mouse model of high‐grade serous ovarian cancer (HGSOC) that recapitulates human tumor–immune interactions. Using combined anti‐PD‐L1/anti‐CD73 immunotherapy, we demonstrate the model's improved biological relevance and enhanced translational value for preclinical immunotherapy testing.

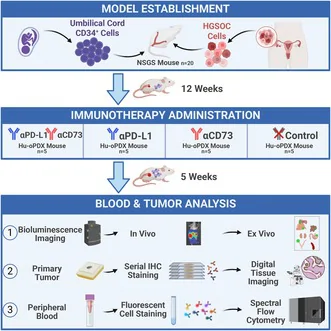

**Abbreviations**
BLI: bioluminescence imaging
FBS: fetal bovine serum
FIGO: International Federation of Gynecology and Obstetrics
GFP: green fluorescent protein
HGSOC: high‐grade serous ovarian cancer
HSC: hematopoietic stem cell
ICI: immune checkpoint inhibitor
ICOS: inducible costimulator
IHC: immunohistochemistry
IM: invasive margin
MACS: magnetic activated cell sorting
MNC: mononuclear cell
NSG: *NOD.Cg‐Prkdc* ^*scid*^ *Il2rg* ^*tm1Wj/*^ *SzJ*
NSGS: *NOD.Cg‐Prkdc* ^*scid*^ *Il2rg* ^*tm1Wjl*^ *Tg (CMV‐IL3, CSF2, KITLG) 1Eav/MloySzJ*
PBS: phosphate‐buffered saline
PDX: patient‐derived xenograft
RBC: red blood cell
RT: room temperature
TAM: tumor‐associated macrophage
TIL: tumor‐infiltrating lymphocyte
TME: tumor microenvironment
Treg: regulatory T cell

## 1 Introduction

High‐grade serous ovarian cancer (HGSOC) is the most common and lethal subtype of ovarian cancer, with a 5‐year survival rate of less than 50% [1]. The implementation of poly(ADP‐ribose) polymerase inhibitors, bevacizumab, and mirvetuximab soravtansine‐gynx as additions to the standard treatment approach has notably improved outcomes of primary and recurrent disease [2, 3]. Despite these advances, HGSOC outcomes are still frequently impaired by treatment resistance and disease recurrence [4, 5], underscoring the urgent need for the exploration of more effective therapeutic approaches.

Although immune‐desert phenotypes are frequently observed, HGSOC can display immunogenic properties, with tumor‐reactive leukocytes detected in a substantial proportion of patients [6], and tumor‐infiltrating lymphocytes (TILs)—particularly CD8^+^ T cells and regulatory T cells (Tregs)—being associated with prognosis and treatment response [7, 8]. These features support the potential of immunotherapy as a viable treatment strategy for HGSOC. Although immunotherapy in the form of immune checkpoint inhibitors (ICIs) has proven effective in several solid cancer types, leading to regulatory approvals [9, 10], clinical trials in HGSOC have consistently yielded disappointing results, with single‐agent ICIs achieving response rates of only 10–15% [11, 12]. One of the key factors responsible for immunotherapy failure in HGSOC is the immunosuppressive network within its tumor microenvironment (TME), which employs multiple immunoinhibitory mechanisms and exhibits remarkable plasticity in response to immunotherapeutic interventions, further complicating treatment efforts [13, 14]. Thus, there is a need for immunotherapy combinations that can surpass these treatment resistance mechanisms of HGSOC. However, no clinical trials examining such approaches have yet demonstrated significant improvements to disease outcome [11, 12, 15].

These trials were often based on experiments in preclinical *in vitro* or *in vivo* models that inadequately represented the TME of HGSOC and the intricate dynamics of its interaction with the human immune system [16, 17, 18, 19, 20, 21]. For example, syngeneic mouse models incorporating murine cancer cell lines implanted into mouse strains with a fully functional murine immune system have commonly been used to evaluate the effectiveness of immunotherapy in HGSOC due to their simplicity and rapid establishment [22, 23], leading to a lack of translational success in clinical applications. To ensure that only biologically relevant immunotherapy combinations advance to human clinical trials, it is imperative to develop preclinical animal models that more accurately reflect the complexity of the disease.

An alternative approach to the above models is to use patient‐derived xenograft (PDX) models, established by implanting tumor material from patients into immunodeficient mice. This type of model effectively preserves the structural and genomic features of the primary tumor, as well as intratumoral heterogeneity and treatment response [24, 25]. While PDX models are typically established via subcutaneous or intraperitoneal injection, orthotopic PDX models, in which patient tumor material is engrafted into the corresponding anatomical site in the model animal, offer the most biologically representative approach as they also preserve the pattern of metastatic spread [26]. However, orthotopic PDX models are less frequently utilized due to their highly immunodeficient nature, requiring specialized handling facilities and procedures, as well as the need for advanced surgical expertise and the reliance on imaging techniques for tumor growth monitoring [27].

Despite facilitating improved representation of the HGSOC TME in the preclinical setting, to effectively simulate the tumor–immune cell interactions critical for disease outcome and immunotherapy success [7, 8, 28], orthotopic PDX models require the co‐engraftment of a human immune system. Humanization of mice can be accomplished through the adoptive transfer of allogenic or autologous leukocyte subsets, most commonly peripheral blood mononuclear cells or T cells. However, these approaches provide only partial humanization, as they fail to capture the full complexity of the human immune system. Furthermore, these leukocytes, having undergone maturation, are more likely to induce graft‐versus‐host disease when placed in a non‐human environment, such as a mouse [29, 30, 31]. In contrast, injection of CD34^+^ human hematopoietic stem cells (HSCs) generates a more comprehensive human immune system, eliminating the risk of graft‐versus‐host disease by resulting in the development of a variety of human leukocytes adapted to murine physiology [32, 33]. Thorough humanization is most consistently established in heavily immunodeficient strains, such as the *NOD.Cg‐Prkdc* ^*scid*^ *Il2rg* ^*tm1Wj/*^ *SzJ* (NSG) and *NOD.Cg‐Prkdc* ^*scid*^ *Il2rg* ^*tm1Wjl*^ *Tg (CMV‐IL3, CSF2, KITLG) 1Eav/MloySzJ* (NSGS) strains [34, 35]. Our group has previously successfully developed HSC‐humanized orthotopic PDX mouse models of HSGOC which have been used to accurately replicate the low efficacy of single‐agent nivolumab in HGSOC [33].

The NSGO‐OV‐UMB1/ENGOT‐OV30 clinical trial, which evaluated combined durvalumab (anti‐PD‐L1) and oleclumab (anti‐CD73) immunotherapy in HGSOC [15], reported a response rate of 4%, which is consistent with the generally low response rates to immunotherapy observed in HGSOC [11, 12]. One motivation for testing this combination in a clinical trial on HGSOC was a successful preclinical study involving treatment of a non‐humanized BALB/c mouse model of murine colorectal cancer with a combination of oleclumab and a murine PD‐1‐binding antibody that, analogous to the mechanism of durvalumab, blocks PD‐1/PD‐L1 interaction. However, in contrast to the results of the NSGO‐OV‐UMB1/ENGOT‐OV30 clinical trial, this preclinical study reported widespread tumor rejection in model mice [21]. To our knowledge, the durvalumab‐oleclumab combination has not been validated in preclinical model systems that more accurately replicate the HGSOC TME or its interactions with the human immune system.

Here, we present the generation of a humanized orthotopic PDX mouse model of HGSOC and its implementation into a preclinical study of combined ICI treatment. The goal of this study was the establishment of a robust, biologically relevant animal model platform for preclinical testing of combination immunotherapies in HGSOC. We aimed to validate the model's translational potential by replicating the conditions of the NSGO‐OV‐UMB1/ENGOT‐OV30 clinical trial of combined durvalumab‐oleclumab treatment and subsequently immunoprofiling blood and tumor sampled from the treated model mice.

## 2 Materials and methods

### 2.1 Ethical considerations concerning experimental model animals

This study was conducted in compliance with the procedures outlined by the Norwegian State Commission for Laboratory Animals, with the approval of the Norwegian Food Safety Authority (Application ID: 25412). Female *NOD.Cg‐Prkdc* ^*scid*^ *Il2rg* ^*tm1Wjl*^ *Tg (CMV‐IL3, CSF2, KITLG) 1Eav/MloySzJ (NSG‐SGM3/NSGS)* mice (aged 6–12 weeks) (cat. no.: 013062; The Jackson Laboratory, Bar Harbor, ME, USA), bred at the animal facility of the University of Bergen, were housed in groups of up to five mice in individually ventilated HEPA‐filtered cages, with regular replacement of autoclaved food, water, bedding and cages.

### 2.2 Acquisition and processing of human tumor material

For this study, an International Federation of Gynecology and Obstetrics (FIGO) stage IIa HGSOC tumor from a treatment‐naïve patient was provided by the Gynecologic Cancer Biobank, Women's Clinic, Haukeland University Hospital, Bergen, Norway. Ethical approval (REK ID: 2014/1907, 2017/612) and written informed consent from the patient were obtained prior to tumor tissue collection (Aug. 2017) and in accordance with the Declaration of Helsinki. The tumor had wild‐type *BRCA1/2* and was later classified as platinum‐resistant. The tumor was sampled during primary cytoreductive surgery and dissociated as previously described [33]. Single cells (henceforth referred to as ‘PDX material’) were cryopreserved at −150°C in a mix of 90% V/V fetal bovine serum (FBS) (cat. no.: F7524; Sigma‐Aldrich, St. Louis, MO, USA) and 10% V/V dimethyl‐sulfoxide (cat. no.: D8418; Sigma‐Aldrich). This PDX material was selected because it exhibited the highest proportion of CD73‐expressing tumor cells among the HGSOC PDX models in our model portfolio (Fig. S1) [36], aligning with the inclusion criterion of the NSGO‐OV‐UMB1/ENGOT‐OV30 clinical trial, according to which over 10% of tumor cells need to be CD73‐positive [15]. We have additionally previously verified the suitability of the chosen PDX material for preclinical immunotherapy evaluation by confirming its responsiveness to both standard‐of‐care and immunotherapeutic intervention in orthotopically xenografted mice (K. Kleinmanns, unpublished data). The PDX material was propagated by two rounds of *in vivo* passaging in immunodeficient mice. Thawed PDX material was implanted into the ovarian bursae of the mice (described in detail in Section 2.6), and the resulting tumors were harvested, processed and cryopreserved in the same manner as the original patient material.

### 2.3 Lentiviral transduction of tumor cells

To enable the *in vivo* monitoring of tumor growth and metastasis in model mice using bioluminescence imaging (BLI), PDX material was transduced using RediFect Red‐FLuc‐GFP lentiviral particles (cat. no.: CLS960003; PerkinElmer, Waltham, MA, USA), containing genes encoding *Luciola italica* firefly luciferase (luc) and green fluorescent protein (GFP) reporters. The transduction was performed according to the following custom protocol. Twice‐passaged PDX material was thawed and washed by centrifugation (400 g, 5 min, room temperature (RT)) in RPMI 1640 cell culture medium (cat. no.: R5886; Sigma‐Aldrich) supplemented with 10% V/V HyClone FBS (cat. no.: SH30071.03HI; Cytiva, Marlborough, MA, USA), 1% V/V L‐glutamine (cat. no.: G7513; Sigma‐Aldrich) and 1% V/V penicillin–streptomycin (cat. no.: P0781; Sigma‐Aldrich) (henceforth referred to as ‘complete RPMI medium’). Cells were counted, seeded into an adherent cell culture plate (cat. no.: 3538; Corning, Corning, NY, USA) at a predetermined number per well, and incubated in complete RPMI medium (18–24 h, 37°C, 5% V/V CO_2_). After incubation, to establish a precise multiplicity of infection (MOI) of 20, cells in prespecified wells were re‐counted—the medium was aspirated, and the cells were detached from the plate after a 5–20 min incubation in 100 μL of accutase (cat. no.: SCR005; Sigma‐Aldrich), neutralized by the addition 1 mL of complete RPMI medium. The medium in the remaining wells was aspirated and replaced with complete RPMI medium containing no FBS and supplemented with Vectofusin‐1 (cat. no.: 130‐111‐163; Miltenyi Biotec, Bergisch Gladbach, Germany) to enhance transduction efficiency. Lentiviral particles were added to the wells at an MOI of 20, followed by spinoculation, which involved centrifugation of the cell culture plate (700 g, 90 min, 32°C) and subsequent incubation (16–24 h, 37°C, 5% V/V CO_2_). After incubation, the plate was centrifuged (400 g, 5 min, RT), and the culture medium containing lentiviral particles was aspirated and replaced with phosphate‐buffered saline (PBS). After centrifugation of the plate (400 g, 5 min, RT) and aspiration of the PBS, cells were detached using accutase as described earlier, transferred to 1.5 mL tubes, and centrifuged (400 g, 5 min, RT). Each cell pellet was subsequently washed by resuspension in PBS and centrifugation (400 g, 5 min, RT). Cells were then orthotopically implanted into mice (described in detail in Section 2.6). Transduced PDX material was propagated *in vivo* for three passages. During each passage, excised tumors were dissociated and cryopreserved as described beforehand, and subsequently thawed PDX material was enriched for high GFP expression prior to the next orthotopic injection using a high‐speed cell sorter (Model SH800; Sony Biotechnology, San Jose, CA, USA).

### 2.4 Isolation of human hematopoietic stem cells

The CD34^+^ human HSCs used in this study were isolated from umbilical cord blood collected during cesarean delivery of a healthy woman with a presumedly healthy pregnancy (Research Biobank for Blood Diseases, Haukeland University Hospital, Bergen, Norway). Ethical approval (REK ID: 2015/1759) and written informed consent from the parents were obtained prior to blood collection (June 2021). The umbilical cord was clamped, and the distal portion of the umbilical vein was immediately punctured with a 12‐gauge needle. A volume of 140 mL of blood was collected in a collection bag containing citrate phosphate dextrose anticoagulant solution (cat. no.: MSC1208DU; Macopharma, Tourcoing, France). Mononuclear cells (MNCs) were then isolated from the umbilical cord blood as follows: The blood was diluted 1 : 1 with sterile, room‐temperature PBS. Diluted blood was laid on top of a density gradient medium ‐ Lymphoprep (cat. no.: 07861; STEMCELL Technologies, Vancouver, Canada), and centrifuged (400 g, 30 min, RT, lowest acceleration, no brakes). Opaque interphases containing MNCs were collected into sterile conical 50 mL tubes and washed by resuspension in a large volume of PBS and subsequent centrifugation (500 g, 5 min, RT). The supernatant was aspirated and the remaining red blood cells (RBCs) were lysed by incubating the cells in 20 mL of 1× RBC Lysis Buffer (cat. no.: TNB‐4300; Cytek Biosciences, Fremont, CA, USA) (8 min, RT, shielded from light). The cell suspension was supplemented with magnetic activated cell sorting (MACS) buffer (2 mM EDTA and 0.5% w/V bovine serum albumin in PBS, pH 7.2) to a total volume of 45 mL and centrifuged (300 g, 10 min, RT). After aspiration of the supernatant, the MNCs were resuspended in 1 mL of CryoStor CS10 (cat. no.: 210502; Biolife Solutions, Bothell, Washington, USA) and cryopreserved at −150°C. On the day of mouse humanization, the MNCs were thawed at 37°C. All MNCs were combined in a new sterile conical 50 mL tube and CD34^+^ HSCs were isolated using MACS with the CD34 MicroBead Kit (cat. no.: 130‐046‐702; Miltenyi Biotec), according to the manufacturer's protocol. The purity of isolated CD34^+^ HSCs was determined by staining an aliquot of the CD34‐enriched cell suspension with a phycoerythrin‐labeled anti‐human‐CD34 antibody (cat. no.: 130‐098‐140; Miltenyi Biotec) and analyzing the cells with an LSRFortessa Cell Analyzer (cat. no.: 649225; Becton, Dickinson and Company, Franklin Lakes, NJ, USA) equipped with the BD FACSDiva Software (v9.0.1, Becton; Dickinson and Company) (Fig. S2). Detailed workflows used for HSC isolation and purity control are available in Protocol S1.

### 2.5 Humanization of model mice

**Fig. 1:**
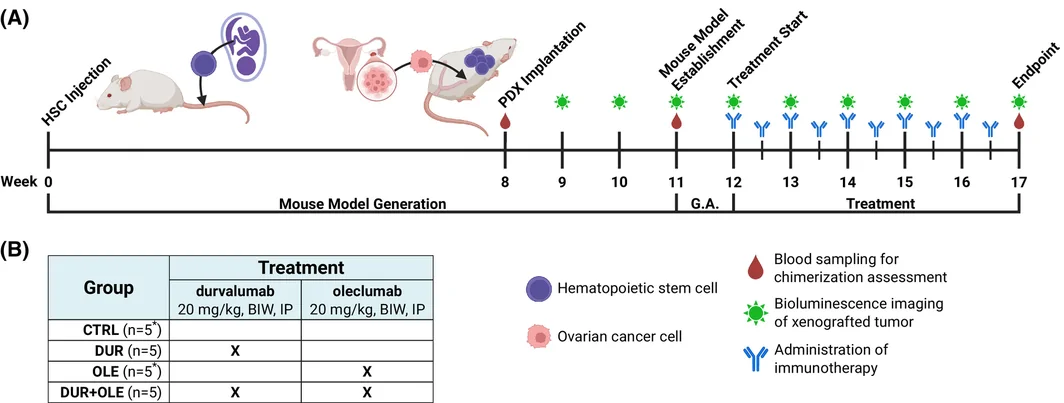
Establishment and application of the mouse model. (A) Timeline for the generation, monitoring, and combination immunotherapy treatment of a murine orthotopic PDX model of treatment‐naïve high‐grade serous ovarian cancer. (B) Distribution of model mice and description of immunotherapy administration across treatment groups. *Initially, five mice were allocated to each treatment group. However, due to the deaths of two mice allocated to the oleclumab‐only group after the first drug injection, one mouse from the control group was reallocated to the oleclumab‐only group, resulting in the oleclumab‐only and control groups consisting of *n* = 4 mice for the remainder of the study. BIW, twice a week; CTRL, control group; DUR + OLE, combination treatment group; DUR, durvalumab‐only group; G.A., group assignment; HSC, hematopoietic stem cell; IP, intraperitoneally; OLE, oleclumab‐only group; PDX, patient‐derived xenograft.

The NSGS mice were humanized by injection of 1.9 × 10^4^ CD34^+^ HSCs suspended in 100 μL of saline into the tail vein (Fig. 1A). All mice were injected with HSCs from the same donor. Chimerism was evaluated by conventional flow cytometry as previously described [33] at three timepoints following HSC injection: 8 weeks (before orthotopic PDX implantation), 11 weeks (prior to treatment initiation), and 17 weeks (at the study endpoint) (Fig. 1A; Fig. S3).

### 2.6 Establishment of high‐grade serous ovarian cancer xenografts in humanized mouse models

To establish the orthotopic PDX mouse model, 8 weeks after HSC injection, GFP^+^/luc^+^ PDX material (passage five) was thawed, and 1.0 × 10^5^ cells were orthotopically injected into the bursa of the right ovary of each of 20 NSGS mice, as described previously [37] (Fig. 1A). Briefly, GFP^+^/luc^+^ cells were prepared for orthotopic injection by resuspension in saline, followed by mixing two parts of the cell suspension with one part of a 1 : 1 mix of Matrigel membrane matrix (cat. no.: 10365602; Corning) and RPMI1640 cell culture medium. After the mice were administered analgesia 5 mg/kg meloxicam (Metacam, 2 mg/mL injection solution, cat. no.: 386860; Boehringer Ingelheim, Ingelheim am Rhein, Germany) and 0.1 mg/kg buprenorphine hydrochloride (Temgesic, 0.3 mg/mL injection solution, cat. no.: 521634; Indivior Inc., North Chesterfield, VA, USA), anesthesia (cat. no.: 002185; Vitusapotek, Oslo, Norway) and ophthalmic lubricant (cat. no.: 006172; Teva Pharmaceuticals, Tel Aviv, Israel), a small incision (~5 mm) was made through the skin and abdominal muscles, mid‐way between the last rib and the iliac crest. The right ovary was grasped by its surrounding fat pad and exteriorized through the incision. With the aid of a microscope with a 10x magnification (SMZ‐171; Motic, Hong Kong, China), a 30‐gauge needle syringe was used to inject 10 μL of the PDX material suspension into the ovarian bursa. Matrigel was allowed to polymerize by delaying the removal of the needle, preventing leakage of the cell suspension. The ovary was carefully repositioned, and the incision was sutured using an absorbable suture (cat. no.: J492G; Agntho's, Lindingö, Sweden). Postoperatively, the animals were given a subcutaneous injection of sterile saline and were allowed to recover in a warm environment before being returned to their home cage.

Growth of the luc^+^ PDX material was monitored weekly by BLI (Fig. 1A). Mice were injected intraperitoneally with 150 mg/kg of D‐luciferin (cat. no.: L‐8220; Biosynth, Staad, Switzerland). Bioluminescent signal was acquired laterally and ventrally 10 min after D‐luciferin administration using the IVIS Spectrum *In Vivo* Imaging System (PerkinElmer). Images were analyzed using the Living Image software (v4.7.3, PerkinElmer).

### 2.7 Treatment

Humanized PDX mice were stratified into four treatment groups containing mice with similar distributions of leukocyte chimerism extent and tumor load, as determined by conventional flow cytometry and BLI, respectively. Based on the NSGO‐OV‐UMB1/ENGOT‐OV30 clinical trial of durvalumab and oleclumab in HGSOC [15], and the preclinical study by Hay et al. [21], the groups and drug dosages were defined as follows: (a) 20 mg/kg durvalumab (*n* = 5); (b) 20 mg/kg oleclumab (*n* = 5); (c) 20 mg/kg of both durvalumab and oleclumab (*n* = 5); (d) untreated control (*n* = 5) (Fig. 1B). Durvalumab (EU No. EU/1/18/1322/002, batch AAUR, AstraZeneca, Cambridge, UK) and oleclumab (cat. no.: HY‐P99039, batches 279777 & 255720; MedChemExpress, Monmouth Junction, NJ, USA) were diluted in sterile saline to concentrations of 3.8 mg/mL and 5.0 mg/mL, respectively, and administered intraperitoneally twice per week for five subsequent weeks. The appearance, activity levels, and food and water intake of the mice were monitored daily, and their weight was measured multiple times a week. Tumor growth was monitored by BLI. Humane endpoints were defined using score sheets and were based on weight loss of over 10% since the most recent weighing, distension of the abdomen caused by ascites, unkempt fur, paleness or lethargy. During the first week of treatment, two mice from the oleclumab‐only group died. In order to preserve statistical power, one mouse from the control group was re‐assigned to the oleclumab‐only group and was administered oleclumab from the second treatment timepoint onward. Consequently, the oleclumab‐only and control groups were reduced to four mice, which continued treatment as planned.

### 2.8 Sample collection and processing

At the end of the study, mice were euthanized according to institutional guidelines due to a combination of factors, including health deterioration. Briefly, mice were anesthetized using sevoflurane. While under anesthesia, a terminal blood sample was collected from the facial vein into an EDTA Microvette (cat. no.: 20.1341.100; Sarstedt, Nümbrecht, Germany), followed by cervical dislocation. The collected blood was processed using Stable‐Lyse2 (cat. no.: STBLYSE2‐250; Smart Tube, Las Vegas, NV, USA) and Stable‐Store2 (cat. no.: STBLSTORE2‐1000; Smart Tube), according to the manufacturer's protocol, and then frozen in cryogenic vials at −80°C. Euthanized mice were dissected ventrally, and the primary tumor characteristics, as well as the presence and extent of metastases, were described macroscopically. The extent of tumor dissemination was additionally assessed *ex vivo* using BLI. Samples of the primary tumors were fixed in 4% V/V formaldehyde (cat. no.: 9713.9010; VWR International, Radnor, PA, USA) for 24 h, then washed using deionized water, and kept in 70% V/V ethanol until paraffinized and sectioned for immunohistochemical (IHC) analysis.

### 2.9 Spectral flow cytometry analysis of the peripheral blood of model mice

Lysed and fixed samples of whole blood were thawed, washed, and stained for spectral fluorescence flow cytometry analysis. A detailed staining workflow (Protocol S2), as well as the antibody panel (Table S1), are available as Supporting Information. Briefly, blood samples were thawed at 4°C, mixed with 0.25 mg/mL DNAse I (cat. no.: DN25; Sigma‐Aldrich) in Dulbecco's PBS containing Ca^2+^ and Mg^2+^ (cat. no.: D8662; Sigma‐Aldrich), and supplemented with CountBright Absolute Counting Beads (cat. no.: C36950; Thermo Fisher Scientific, Waltham, MA, USA). Samples were washed in PBS and filtered through the 40 μm mesh in the cap of 5 mL round‐bottom tubes (cat. no.: 352235; Corning). Pelleted cells were incubated in a solution of human Fc‐receptor blocking agent (cat. no.: 130‐059‐901; Miltenyi Biotec,) and anti‐mouse‐CD16/CD32 monoclonal antibody (cat. no.: 16‐0161‐82; Thermo Fisher Scientific), and then stained with the antibody mix defined in Table S1. Stained cells were washed twice with a mix of 2% V/V FBS in PBS and acquired on an ID7000 Spectral Cell Analyzer (LE‐ID7000C; Sony Biotechnology) equipped with ID7000 Software (v2.0.2.17121; Sony Biotechnology). Single‐cell data were processed using FlowJo software (v10.10.0; Becton, Dickinson and Company) (Fig. S4). Absolute leukocyte quantities were calculated using blood volume estimations based on Counting Bead data. The composition of the human leukocyte pool in each sample was calculated by dividing the number of cells of a specific subset by the total number of human leukocytes in that sample.

### 2.10 Immunohistochemical staining of primary mouse tumors

Immunohistochemical staining of serial sections of paraffinized primary tumor tissue was performed according to the workflow described in Protocol S3. In short, paraffinized tumor sections with a thickness of 3 μm were deparaffinized in xylene and rehydrated in a graded ethanol series. After antigen retrieval at a pH of 9.0, blocking of endogenous peroxidase activity was performed. Next, tissues were incubated for 45‐60 min in a blocking solution of 3% w/V bovine serum albumin (cat. no.: 10735086001; Merck, Darmstadt, Germany) to mitigate nonspecific antibody binding. After two washes, primary antibodies targeting human CD45 (cat. no.: 14‐9457‐82; Thermo Fisher Scientific), CD20 (cat. no.: 555677; Becton, Dickinson and Company), CD3 (cat. no.: ab17143; Abcam, Cambridge, UK), CD8 (cat. no.: 372902; BioLegend, San Diego, CA, USA) and FoxP3 (cat. no.: 14‐4777‐82; Thermo Fisher Scientific) were individually applied to five serial sections of each primary PDX tumor and left to incubate overnight at 4°C. Antibody details are provided in Table S2. The next day, primary antibodies were washed off, and appropriate horseradish‐peroxidase‐labeled secondary antibodies were applied to the tissues. After the secondary antibody was washed off, antigen localization was visualized via a 3,3′‐diaminobenzidine reaction. Stained tissues were washed, counterstained with hematoxylin (cat. no.: S3301; Agilent, Santa Clara, CA, USA), and mounted using an automated coverslipper (Model 4740, Sakura, Torrance, CA, USA). The immunophenotype [38] of all primary tumors was microscopically evaluated as immune‐excluded by a trained pathologist, as leukocytes were present within the tumors, but confined to the stroma surrounding tumor cell foci.

### 2.11 Digital tissue analysis

Stained primary tumor slides were digitally scanned at a 400x magnification using a slide scanner (Model BX61VSF; Olympus, Tokyo, Japan) equipped with Olympus VS‐ASW software (v2.9.2; Olympus). Digital cell segmentation and quantification were performed on the high‐resolution digital scans using QuPath software (v0.5.1) [39]. Briefly, based on previously published approaches [40, 41], areas with the highest density of leukocyte infiltration were selected for annotation. Due to the immune‐excluded nature of the tumors, annotations were drawn along the invasive margin (IM) of tumors, symmetrically extending 200 μm from each side of the tumor‐stroma border of the IM. Automatic staining vector estimation and optical‐density‐based cell segmentation were used for the enumeration of marker‐positive leukocytes. A detailed overview of positive cell detection parameters is available in Table S3. For each tumor and marker, the density of marker‐positive TILs per mm^2^ was calculated by dividing the total number of positive TILs by the total combined size of the annotated area.

### 2.12 Statistical analyses

Statistical analyses were performed using GraphPad Prism (v10.4.1, GraphPad Software, San Diego, CA, USA). Primary tumor volumes at endpoint, quantities of leukocytes per volume of blood, relative leukocyte abundances, and TIL densities were compared between treatment groups. For each sample group (*n* = 4 or *n* = 5), the Shapiro–Wilk test was used to assess the normality of the datasets. Inter‐group comparisons were performed using the Kruskal‐Wallis test with Dunn's multiple comparison correction. Ratios of CD8^+^ and FoxP3^+^ TIL densities were compared between groups using one‐way ANOVA with Tukey's multiple comparison correction. Correlations between TIL density and tumor burden (volume) were evaluated as follows: for each staining marker, measurements of leukocyte density were either stratified according to treatment group or considered as one group. After assessment of dataset normality using the Shapiro–Wilk test, Pearson or Spearman rank correlation tests were performed. Correlation plots were modeled using simple linear regression. Due to the small group size and large confidence intervals, formal outlier analyses were not feasible. Statistical significance was defined as p < 0.05.

## 3 Results

### 3.1 Successful establishment of a humanized PDX mouse model for preclinical testing of combination immunotherapy in HGSOC

We successfully implemented the mouse model establishment workflow described by Kleinmanns et al. [33], utilizing HSCs and tumor material from unique donors to create a humanized PDX model for preclinical testing of combination immunotherapy in HGSOC (Fig. 1A). Despite injecting significantly fewer CD34^+^ HSCs per mouse compared with Kleinmanns *et al*., chimerism analysis of peripheral blood samples obtained at multiple timepoints after the injection demonstrated stable HSC engraftment and sustained development of human lymphocyte populations in the experimental mice (Table S4). Following orthotopic implantation of PDX material in week eight, BLI conducted during Weeks 9‐11 revealed detectable tumor signal localized to the ovary in all mice, confirming successful orthotopic tumor engraftment (Table S5). Following confirmation of sufficient chimerism and stable PDX engraftment, the 20 experimental mice were evenly allocated into four treatment groups, ensuring comparable mean chimerism levels and bioluminescence signal intensities across groups (Fig. 1B). All mice receiving combination therapy demonstrated good tolerance to the treatment, with no observable deterioration in their condition compared with the other treatment and control groups.

Two mice from the oleclumab‐only group died three days after the first drug administration. No signs of advanced disease, weight loss, or behavioral changes were observed prior to the animals' deaths. Drug‐related side effects and toxicity were ruled out, as the mice were healthy during the two days following injection, and because none were observed in the other animals receiving oleclumab, either by itself or in combination with durvalumab, during the following five weeks of treatment. Therefore, the cause of the mice's death was most likely human error related to drug administration.

### 3.2 The established HGSOC PDX tumor model resists growth inhibition by immunotherapy

**Fig. 2:**
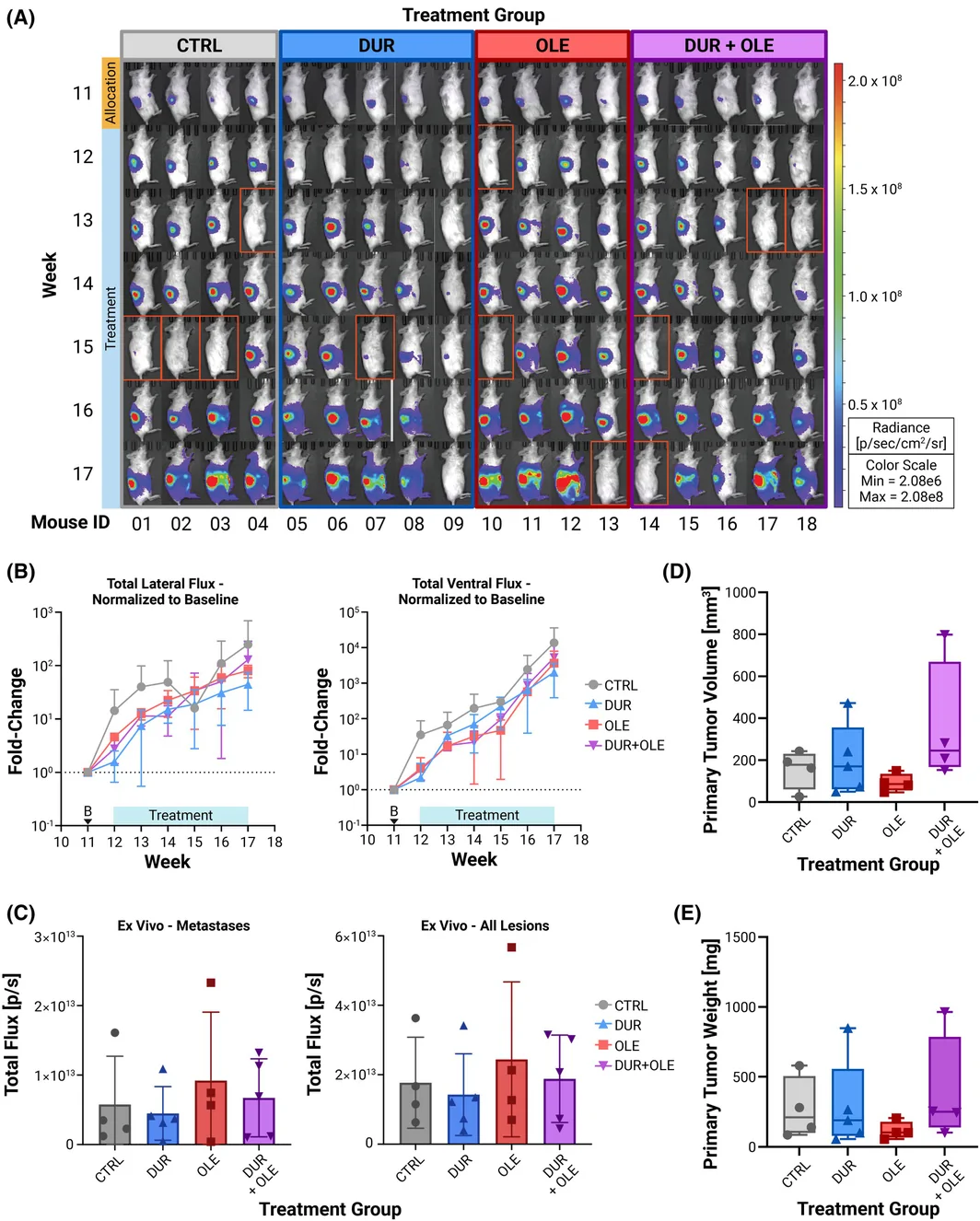
Monitoring of tumor burden in PDX‐implanted experimental mice. (A) Longitudinal overview of weekly bioluminescence imaging results for PDX‐implanted mice (*n* = 18) in the lateral position. Each column represents an individual mouse. Only images from baseline (week 11) through endpoint (week 17) are shown (scale bar 2 × 10^6^−2 × 10^8^ p/s/cm^2^/sr). Mice outlined with an orange border displayed inexplicably low bioluminescence at the specified timepoint, even after luciferin re‐injection. These data were excluded from further analyses. Bioluminescence images of the mice taken ventrally are displayed in Fig. S5A. Full data on the total lateral flux are available in Table S5. (B) Average total lateral (left graph) and ventral (right graph) photon flux in each treatment group during treatment, relative to baseline (marked by a ‘B’ on the *X*‐axis). The error bars represent one standard deviation from the mean. (C) Average *ex vivo* bioluminescence signal intensities of metastatic lesions (left) and total tumor burden (right) across all treatment groups. The signal intensity of the metastatic lesions was calculated by subtracting the signal intensity of the primary tumor from that of all lesions. (D) Comparison of primary tumor volume and (E) primary tumor weight at the end of the study between treatment groups. In (D) and (E), only the primary tumor was included in tumor burden assessment due to the small size of the metastatic lesions. In (B–E), no inter‐group differences were significant. Tumor measurement data are available in Table S6. *P*‐values of inter‐group differences were calculated using the Kruskal‐Wallis test with Dunn's multiple comparison correction. Box and whisker plots represent the median (central line), interquartile range (IQR) (box), and minimum and maximum values within 1.5 * IQR out from the IQR (whiskers). CTRL, control group; DUR + OLE, combination treatment group; DUR, durvalumab‐only group; OLE, oleclumab‐only group; PDX, patient‐derived xenograft.

Longitudinal weekly BLI of the xenografted PDX material showed consistent tumor growth and progressive disease in all mice (Fig. 2A, Fig. S5A). Although the average bioluminescent signal was marginally higher in the control group than in all treatment groups throughout the observation period (Fig. 2B, Fig. S5B), this difference was not statistically significant. Furthermore, tumor growth kinetics were similar across all four groups, with none of the treatments resulting in a sustained reduction in bioluminescence signal (Fig. 2B, Fig. S5B). At the end‐of‐study necropsy, *ex vivo* BLI imaging showed no significant differences in total tumor burden across treatment groups (Fig. 2C). Measurements of excised primary tumor volume showed no statistically significant difference in tumor burden between the treatment groups (Fig. 2D, Table S6), nor did measurements of primary tumor weight (Fig. 2E, Table S6). The extent of metastatic dissemination was also similar across groups, with nearly all mice exhibiting abdominal carcinomatosis, with visible metastatic lesions on the omentum, peritoneal wall, and/or diaphragm (Fig. S6, Table S7).

### 3.3 The administration of immunotherapy does not enhance intratumoral infiltration of immune cells in the established HGSOC PDX model

**Fig. 3:**
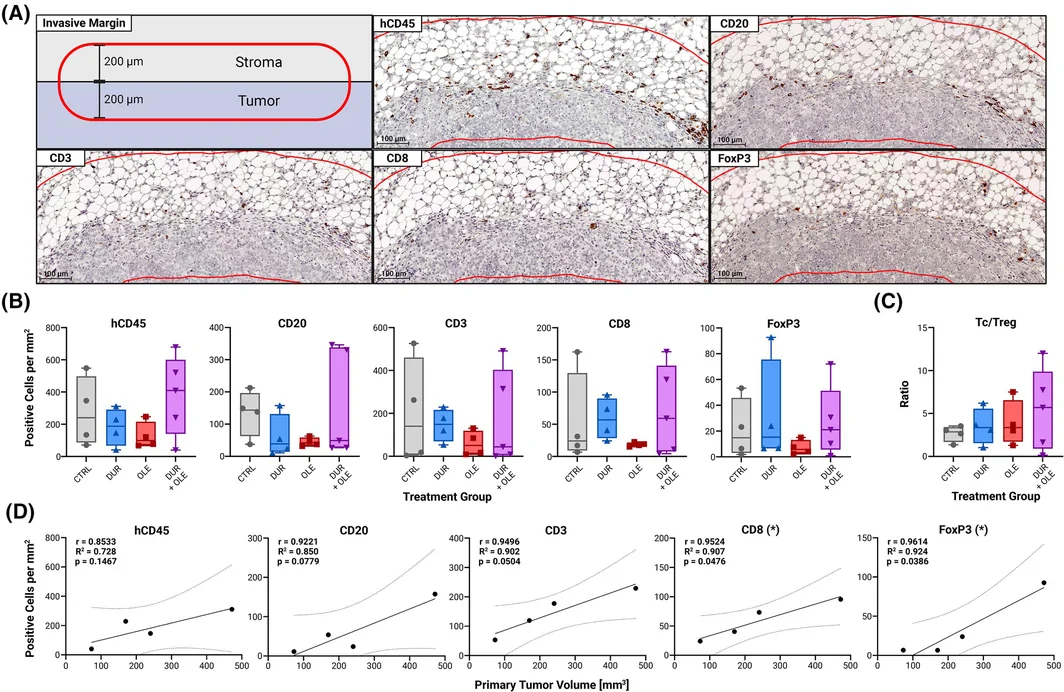
Intratumoral leukocyte densities determined by immunohistochemistry (IHC) and digital analysis using the QuPath software. (A) Representative images of tumor areas rich in leukocytes selected for positive cell quantification. The top‐left image is a schematic representation of the annotation method: By tracing the tumor‐stroma border of the invasive margin with a 400‐μm‐thick brush tool, an invasive tumor margin of symmetrical intra‐stromal and intratumoral depths of 200 μm was delineated. The remaining images display serial primary PDX tumor sections stained with antibodies targeting the leukocyte marker specified in the upper‐left corner of each image. Antibody details are provided in Table S2. (B) Comparison of intratumoral leukocyte densities between treatment groups. Full data on intratumoral leukocyte density are available in Table S8. (C) Inter‐group comparison of the ratios of CD8^+^/cytotoxic (Tc) and FoxP3^+^/regulatory (Treg) TIL densities. (D) Plots depicting correlations between tumor burden at the end of the study and the densities of intratumoral marker‐positive leukocytes for the group of mice treated with durvalumab (*n* = 4). Significant correlations (*P* < 0.05) are marked with an asterisk (*) in the graph title. All correlation plots are displayed in Fig. S8, and full correlation data are available in Table S9. Depending on the results of a Shapiro–Wilk normality test, either the Pearson or Spearman correlation test was used to determine the *P*‐value for each dataset. In (B) and (C), no inter‐group differences were significant. *P*‐values of inter‐group differences were calculated using the Kruskal‐Wallis test with Dunn's multiple comparison correction. Box and whisker plots represent the median (central line), interquartile range (IQR) (box), and minimum and maximum values within 1.5 * IQR out from the IQR (whiskers). In (D), the dashed lines represent the 95% confidence interval of the best‐fit line. CTRL, control group; DUR + OLE, combination treatment group; DUR, durvalumab‐only group; OLE, oleclumab‐only group; PDX, patient‐derived xenograft; *r*, Pearson's correlation coefficient; Tc, cytotoxic (CD8^+^) T cell; Treg, regulatory (FoxP3^+^) T cell.

To assess the effectiveness of the combined durvalumab‐oleclumab treatment in promoting leukocyte infiltration into the HGSOC PDX tumor and compare it with the impact of the individual immunotherapeutic agents, all excised primary tumors were processed for paraffin embedding, sectioned, and immunohistochemically stained. The staining targeted markers for total human leukocytes (hCD45), B cells (CD20), and the intended targets of durvalumab and oleclumab immunotherapy: total T cells (CD3), cytotoxic T cells (CD8), and Tregs (FoxP3). Brightfield microscopy scans of the stained PDX tumor sections showed that, in all tumors, marker‐positive cells were predominantly localized to the outer, stromal region of the IM, with scarce leukocyte infiltration into tumor‐rich areas (Fig. S7). Consequently, all tumors were categorized as immune‐excluded [38]. The most densely infiltrated regions of the IMs were selected for enumeration of human leukocytes using automated detection of marker‐positive cells (Fig. 3A). The analysis showed no significant differences in intratumoral leukocyte infiltration among the treatment groups (Fig. 3B, Table S8). Similarly, the median ratios of cytotoxic to regulatory T cells showed no significant variation between the groups (Fig. 3C). To evaluate the relationship between leukocyte infiltration and tumor burden in the context of each treatment, we examined correlations between primary tumor volume at the end of the study and the densities of marker‐positive leukocytes in the IM. No significant correlations or trends were observed in the combination treatment group or for most markers in the other groups. The only exception was the durvalumab monotherapy group, in which increased densities of CD8^+^ cells and FoxP3^+^ cells were significantly positively correlated with larger tumor burden (Fig. 3D, Fig. S8, Table S9).

### 3.4 T‐cell abundances in the peripheral blood of the humanized HGSOC PDX model mice are not altered by immunotherapy

**Fig. 4:**
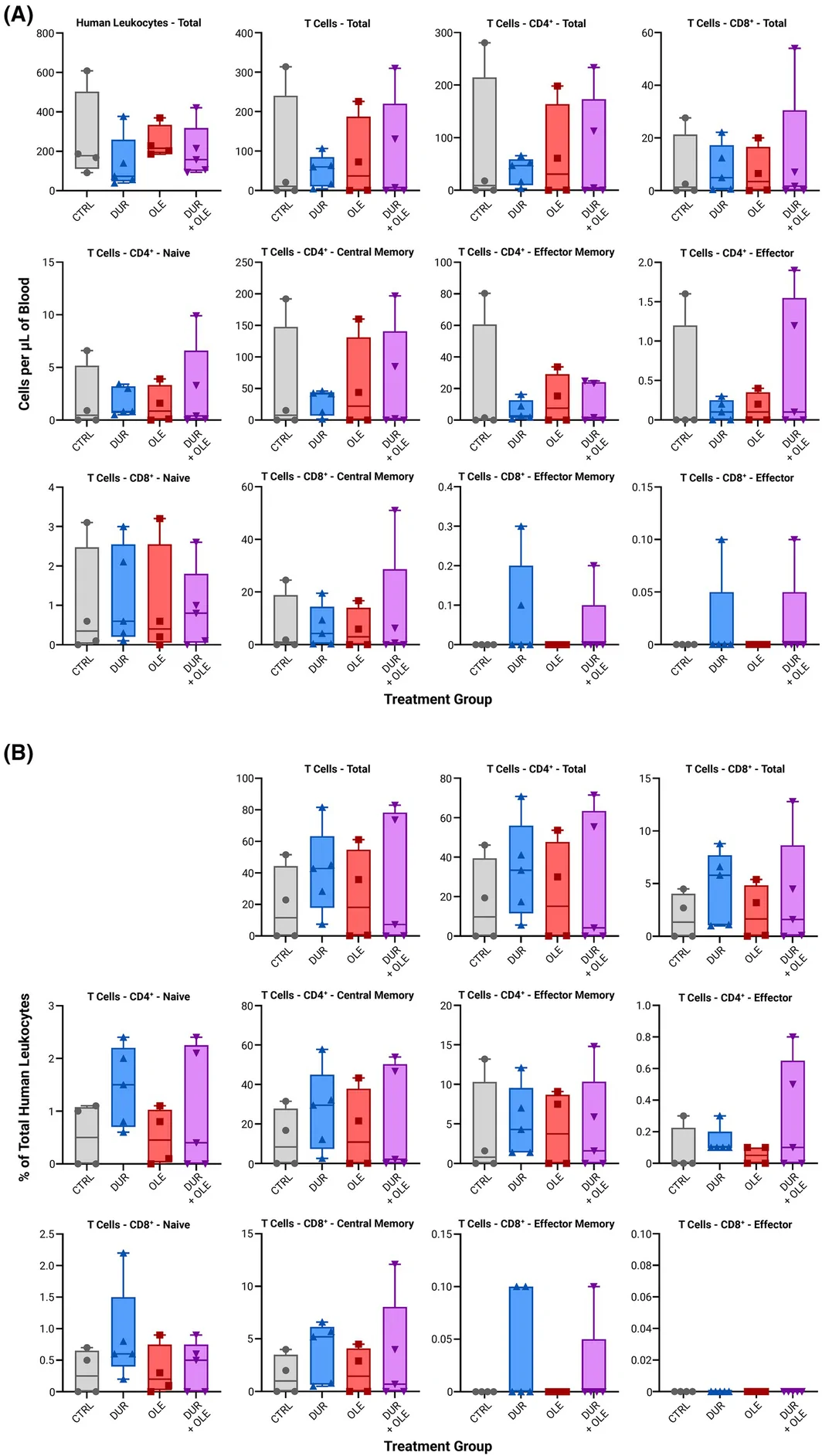
Results of the spectral flow cytometry analysis of blood samples taken at the end of the study (*n* = 18). Data on the human leukocyte counts are available in Table S10. (A) Comparison of total human leukocyte and T‐cell subset frequencies per volume of blood between treatment groups. Data on the human leukocyte counts per μL of blood are available in Table S11. (B) Distribution of T‐cell subsets across treatment groups, relative to total human leukocytes. Data on the abundances of human leukocyte subsets relative to total human leukocytes in the blood are available in Table S12. No inter‐group differences were significant. *P*‐values of inter‐group differences were calculated using the Kruskal‐Wallis test with Dunn's multiple comparison correction. Box and whisker plots represent the median (central line), interquartile range (IQR) (box), and minimum and maximum values within 1.5 * IQR out from the IQR (whiskers). CTRL, control group; DUR + OLE, combination treatment group; DUR, durvalumab‐only group; OLE, oleclumab‐only group.

Comprehensive characterization of the T‐cell repertoire in peripheral blood collected from experimental mice at the end of the study was conducted using spectral flow cytometry. This analysis aimed to identify differences in the absolute and relative abundances of T‐cell subsets across treatment groups (Fig. S4A; Table S10). The implementation of fluorescent counting beads at the start of the staining workflow enabled the precise assessment of the blood volume underlying each dataset, allowing for the determination of absolute leukocyte counts per microliter of blood. In all samples, CD4^+^ T cells exhibited higher absolute abundance than CD8^+^ T cells, with central memory cells being the most prevalent subset within both T‐cell types (Fig. 4A, Table S11). While effector memory cells constituted the second most abundant CD4^+^ T‐cell subset in nearly all samples, precursor effector CD4^+^ T cells were rare (Table S11). Effector and effector memory CD8^+^ T cells were extremely rare, with fewer than one cell per microliter of blood, even in samples with a high overall abundance of CD8^+^ T cells (Table S11). No inter‐group differences in absolute leukocyte counts were observed (Fig. 4A). The relative abundances of T‐cell subsets, normalized to total leukocytes, mirrored the patterns seen in absolute counts and showed no significant immunotherapy‐induced changes (Fig. 4B; Table S12). We attempted to assess T‐cell exhaustion by measuring PD‐L1 expression with a previously validated antibody; however, the combination of negligible PD‐L1 expression and low T‐cell counts in several samples precluded reliable analysis (Fig. S4A–C; Table S13).

## 4 Discussion

Optimizing translation of preclinical research into clinical practice is essential for improving HGSOC outcomes. We build directly upon our experience developing immunocompetent mouse models, including the first HSC‐humanized orthotopic PDX models of HGSOC, which enabled the characterization of tumoral and immunological responses to nivolumab [33]. The NSGO‐OV‐UMB1/ENGOT‐OV30 trial of combined durvalumab and oleclumab aimed to address the limited efficacy of single‐agent immunotherapy in HGSOC but resulted in poor response rates [15]. This stands in contrast to the findings from the preclinical study of the drug combination, which demonstrated robust tumor rejection [21]. To investigate combinatorial immunotherapy in the context of patient‐derived HGSOC, we sought to test the durvalumab‐oleclumab regimen in our HSC‐humanized orthotopic HGSOC PDX model system. Since intratumoral CD73 positivity was a key trial inclusion criterion, we selected the patient material with the highest tumor cell CD73 expression in our PDX portfolio to construct the model for this study. Determining whether the resulting tumor maintained high CD73 expression throughout its growth in the mice would have benefited treatment response evaluation. Unfortunately, due to small tumor size (Fig. 2A) and the ethical obligation to minimize animal suffering, a pretreatment biopsy was infeasible.

Hay et al.'s study of the durvalumab‐oleclumab regimen in a syngeneic mouse model of colorectal cancer demonstrated that simultaneous inhibition of PD‐1/PD‐L1 interaction and adenosine generation induced tumor rejection in 60% of subcutaneous‐tumor‐bearing mice [21]. Conversely, this dual‐targeting strategy failed in clinical trials for ovarian, colorectal, lung, and pancreatic cancer [15, 42]. Hay *et al.'*s preclinical study modeled a murine immune response to murine cancer rather than a human immune response to human malignancies, which may limit clinical translatability. To address this, we used HSC‐humanized NSGS mice orthotopically engrafted with patient‐derived HGSOC tumors to evaluate durvalumab‐oleclumab treatment.

The lack of therapeutic benefit from all treatments in our study aligns with findings that HGSOC often exhibits immunotherapy resistance [11, 12], and with the modest response observed in the NSGO‐OV‐UMB1/ENGOT‐OV30 trial [15]. PD‐L1 expression was confirmed in our PDX material, but did not, in line with the NSGO‐OV‐UMB1/ENGOT‐OV30 trial, prove to be a predictive biomarker in our study. The first evidence of improved survival among HGSOC patients receiving immunotherapy emerged from the ENGOT‐OV65 trial, in which pembrolizumab was combined with paclitaxel, with or without bevacizumab [43]. Notably, the survival benefit was confined to patients with PD‐L1‐positive tumors, highlighting the potential of biomarker‐guided patient stratification to increase the therapeutic impact of ICIs. Paclitaxel possesses immunomodulatory properties and has therefore been considered a suitable combination partner for ICIs to overcome resistance. Whether the combination exerts same effect in PD‐L1‐enriched humanized HGSOC PDX models remains to be determined.

Immunoprofiling of the primary PDX tumors showed no significant differences in TIL densities among treatment groups, with all xenografts generating an immune‐excluded tumor, regardless of treatment. Multiple preclinical studies in humanized HGSOC‐bearing mice, including our previous work, have reported intratumoral T‐cell infiltration following PD‐1/PD‐L1 interaction inhibition. However, none have contextualized their findings with regard to the spatial distribution of T cells within the TME, that is, the tumor's immunophenotype. As these studies have observed a wide variety of treatment responses, it is clear that this was a critical oversight in evaluating their models' predictive power [31, 33, 44]. This, in concert with our current study's results and previous findings that HGSOC immunophenotype is prognostic of disease outcome and predictive of immunotherapy efficacy [45, 46], emphasizes the need for its evaluation, e.g., using simple IHC, before implementing HGSOC into a PDX model or reaching a clinical decision on immunotherapy administration.

Paradoxically, we observed a significant positive correlation between tumor burden and intratumoral densities of both CD8^+^ cytotoxic T cells and FoxP3^+^ Tregs in the durvalumab‐only group (Fig. 3D). We hypothesize that durvalumab initially enhanced CD8^+^ TIL density by facilitating their recruitment and subsequent proliferation. The influx of CD8^+^ TILs triggered a reactive expansion and immunosuppressive capacity increase of the intratumoral FoxP3^+^ Tregs, counteracting immune‐mediated tumor growth suppression. This type of Treg‐mediated immunosuppression during PD‐1/PD‐L1 interaction inhibition has been described in melanoma by Geels *et al*. They demonstrated that interleukin‐2 secreted by non‐exhausted CD8^+^ T cells induces inducible costimulator (ICOS) expression in Tregs, whose ligation by ICOS‐ligand in the TME stimulates activation, proliferation, and expression of T‐cell exhaustion mediators [47]. Interestingly, correlations between tumor size and TIL density were non‐significant and negative in the durvalumab‐oleclumab combination group. Adenosine induces vasodilation in hypoxic environments, such as within a solid tumor, and is critical in conditions like myocardial ischemia [48]. In our study, oleclumab monotherapy resulted in the lowest median intratumoral density of total human leukocytes, implying that oleclumab‐mediated inhibition of adenosine generation may have impaired leukocyte extravasation into tumor tissue via vasoconstriction. This would restrict the anti‐tumor immune response in the durvalumab‐oleclumab treatment group to the leukocytes already present in the TME. Durvalumab's inability to overcome Treg‐mediated immunosuppression could have consequently led to the deceleration of mutually regulated CD8^+^ and FoxP3^+^ TIL proliferation, allowing tumor growth to surpass TIL expansion, resulting in diminished TIL density.

In the peripheral blood of the experimental mice, we observed an accumulation of central memory CD8^+^ T cells, alongside an absence of effector memory and effector CD8^+^ T cells across all treatment groups. This suggests that the T cells, while capable of recognizing and infiltrating HGSOC, were unable to achieve full activation or maintain functionality. Furthermore, Tregs can prevent effector T‐cell activation and regeneration while maintaining central memory T‐cell abundance [49], strongly implicating them as a primary driver of immunosuppression in our model.

While the consistency in the development of an immune‐excluded immunophenotype and comprehensive immunotherapy resistance underscore the stability of our model, as well as its portrayal of the adaptive immunosuppression within the HGSOC TME, it also accentuates a limitation of our study regarding the lack of diversity within the study cohort. The use of a single cord blood donor and a single tumor material donor effectively restricts this study to a single‐patient preclinical trial, limiting our model's generalizability to the broader HGSOC patient population. Future research will therefore focus on developing a diverse preclinical model portfolio incorporating HSCs and HGSOC tumors from various donors, capturing different patient characteristics, disease stages, and immunophenotypes. Our model's value could be further enhanced by using tumor material and bone marrow HSCs from the same patient to construct more personalized ‘avatar’ models. Furthermore, although the allogeneity of the two donors of the engrafted materials did not facilitate immunological tumor destruction in this model, as supported by the continuous tumor growth in all mice (Fig. 2B, Fig. S5B, Table S5), the utilization of syngeneic HSCs and tumor cells for humanized mouse model establishment will circumvent this potentially confounding negative influence on tumor development.

## 5 Conclusions

This proof‐of‐concept study introduces a humanized orthotopic PDX mouse model of HGSOC that effectively replicates the morphology, immune contexture, and immunotherapy resistance of the immunosuppressive HGSOC TME. We demonstrate the model's translational potential and feasibility in analyses of immune suppression mechanisms and tumor‐immune interactions within the TME of HGSOC. Our model offers a platform for preclinical evaluation of combination immunotherapies in HGSOC, enhancing the potential for successful translation of promising preclinical results to clinical trials and improvements in patient care.

## Conflict of interest

LB reports leadership roles in Onkologisk Forum between 2018 and 2022, and in the Nordic Society of Gynaecological Oncology (NSGO) and NSGO—Clinical Trials Unit between 2021 and 2024; receipt of a research grant for a researcher‐initiated trial in ovarian cancer from AstraZeneca; and receipt of honoraria for holding lectures from GlaxoSmithKline. LCVT reports receipt of financial support for a researcher‐initiated trial from AstraZeneca; and receipt of personal fees from Bayer, Eisai Co., and AstraZeneca. EMC reports share ownership in, and chairing the board of KinN Therapeutics AS.

## Author contributions

LT, LB, and KK contributed to the study conceptualization. LT, LB, CFT, PA, RE, DEC, and KK contributed to the experiment design. LT, LB, CFT, PA, RE, DEC, LAA, EMC, and KK contributed to the provision of experimental resources. LT, MRL, CFT, PA, and KK contributed to the data collection. LT, LB, MRT, PA, RE, and KK contributed to the data curation. LT, MRL, PA, DEC, KK contributed to the data analysis and statistics. LT, LB, RE, and KK contributed to the data interpretation. LT, LB, LCVT, and KK contributed to the manuscript writing and editing. LB, LAA, LCVT, EMC, and KK contributed to the supervision. LB and KK contributed to the project administration. LB, LAA, and EMC contributed to the funding acquisition. All authors reviewed the manuscript.

## Supporting information


**Fig. S1.** CD73 expression profiles of the constituents of the dissociated PDX material used in this study.
**Fig. S2.** Gating strategy used for assessing the purity of samples enriched with human CD34+ hematopoietic stem cells from umbilical cord blood.
**Fig. S3.** Representative gating strategy for the assessment of blood chimerism in mice injected with human hematopoietic stem cells.
**Fig. S4.** Key elements of the workflow used for the analysis of endpoint blood samples by spectral flow cytometry.
**Fig. S5.** Longitudinal overview of weekly bioluminescence imaging results for PDX‐implanted experimental mice in the ventral position and of the absolute photon flux.
**Fig. S6.** *Ex vivo* evaluation of intraabdominal tumor dissemination using BLI, presented for individual mice.
**Fig. S7**. Light microscopy images of representative areas of the primary PDX tumor displaying prominent accumulation of human leukocytes in the invasive margin.
**Fig. S8.** Correlation plots showing associations between tumor burden at the end of the study and densities of intratumoral marker‐positive leukocytes.
**Table S1.** Antibody panel used for the characterization of leukocytes in the blood samples from the experimental mice using spectral flow cytometry.
**Table S2.** List of antibodies used for the immunohistochemical staining of primary patient‐derived xenograft tumor sections.
**Table S3.** Overview of the positive cell detection parameters used for the enumeration of leukocytes in primary PDX tumor sections.
**Table S4.** Results of the chimerism assessments of mouse blood during model establishment and at the end of the study.
**Table S5.** Total lateral and ventral photon flux measured weekly during weeks 9 through 17 after injection of hematopoietic cells into the experimental mice.
**Table S6.** Dimensions of primary patient‐derived xenograft tumors measured at the end of the study.
**Table S7.** The extent of visible metastatic dissemination at the end of the study.
**Table S8.** Results of the digital analysis of primary patient‐derived xenograft tumor sections.
**Table S9.** Parameters of the correlations between tumor burden at the end of the study and densities of intratumoral marker‐positive leukocytes for all samples combined and each treatment group individually.
**Table S10.** Results of the spectral flow cytometry analysis of leukocytes in the blood samples from the experimental mice ‐ human leukocyte counts.
**Table S11.** Results of the spectral flow cytometry analysis of leukocytes in the blood samples from the experimental mice ‐ human leukocyte counts per μL of blood.
**Table S12.** Results of the spectral flow cytometry analysis of leukocytes in the blood samples from the experimental mice ‐ human leukocyte abundances relative to total human leukocytes.
**Table S13.** PD‐L1 expression levels on T cells and relative abundances of PD‐L1‐positive T cells in mouse blood taken at the end of the study.
**Protocol S1.** Isolation of Hematopoietic Stem Cells from Umbilical Cord Blood.
**Protocol S2.** Preparation of Blood Samples for Spectral Flow Cytometry Analysis.
**Protocol S3.** Immunohistochemical Staining of Primary Mouse Tumor Sections.

## Data Availability

The data that support the findings of this study are available in the Supporting Information of this article.
