# Supplementary material for: Establishment of a humanized patient‐derived xenograft mouse model of high‐grade serous ovarian cancer for preclinical evaluation of combination immunotherapy
Source: Mol Oncol. 2026 Mar 14:10.1002/1878-0261.70231. Online ahead of print. doi: 10.1002/1878-0261.70231 (PMC13398843; doi:10.1002/1878-0261.70231)
Supplement: Supplementary file 1 — Fig. S1. CD73 expression profiles of the constituents of the dissociated PDX material used in this study. Fig. S2. Gating strategy used for assessing the purity of samples enriched with human CD34+ hematopoietic stem cells from umbilical cord blood. Fig. S3. Representative gating strategy for the assessment of blood chimerism in mice injected with human hematopoietic stem cells. Fig. S4. Key elements of the workflow used for the analysis of endpoint blood samples by spectral flow cytometry. Fig. S5. Longitudinal overview of weekly bioluminescence imaging results for PDX‐implanted experimental mice in the ventral position and of the absolute photon flux. Fig. S6. Ex vivo evaluation of intraabdominal tumor dissemination using BLI, presented for individual mice. Fig. S7. Light microscopy images of representative areas of the primary PDX tumor displaying prominent accumulation of human leukocytes in the invasive margin. Fig. S8. Correlation plots showing associations between tumor burden at the end of the study and densities of intratumoral marker‐positive leukocytes. Table S1. Antibody panel used for the characterization of leukocytes in the blood samples from the experimental mice using spectral flow cytometry. Table S2. List of antibodies used for the immunohistochemical staining of primary patient‐derived xenograft tumor sections. Table S3. Overview of the positive cell detection parameters used for the enumeration of leukocytes in primary PDX tumor sections. Table S4. Results of the chimerism assessments of mouse blood during model establishment and at the end of the study. Table S5. Total lateral and ventral photon flux measured weekly during weeks 9 through 17 after injection of hematopoietic cells into the experimental mice. Table S6. Dimensions of primary patient‐derived xenograft tumors measured at the end of the study. Table S7. The extent of visible metastatic dissemination at the end of the study. Table S8. Results of the digital analysis of primary patient [file MOL2-9999-0-s001.docx]

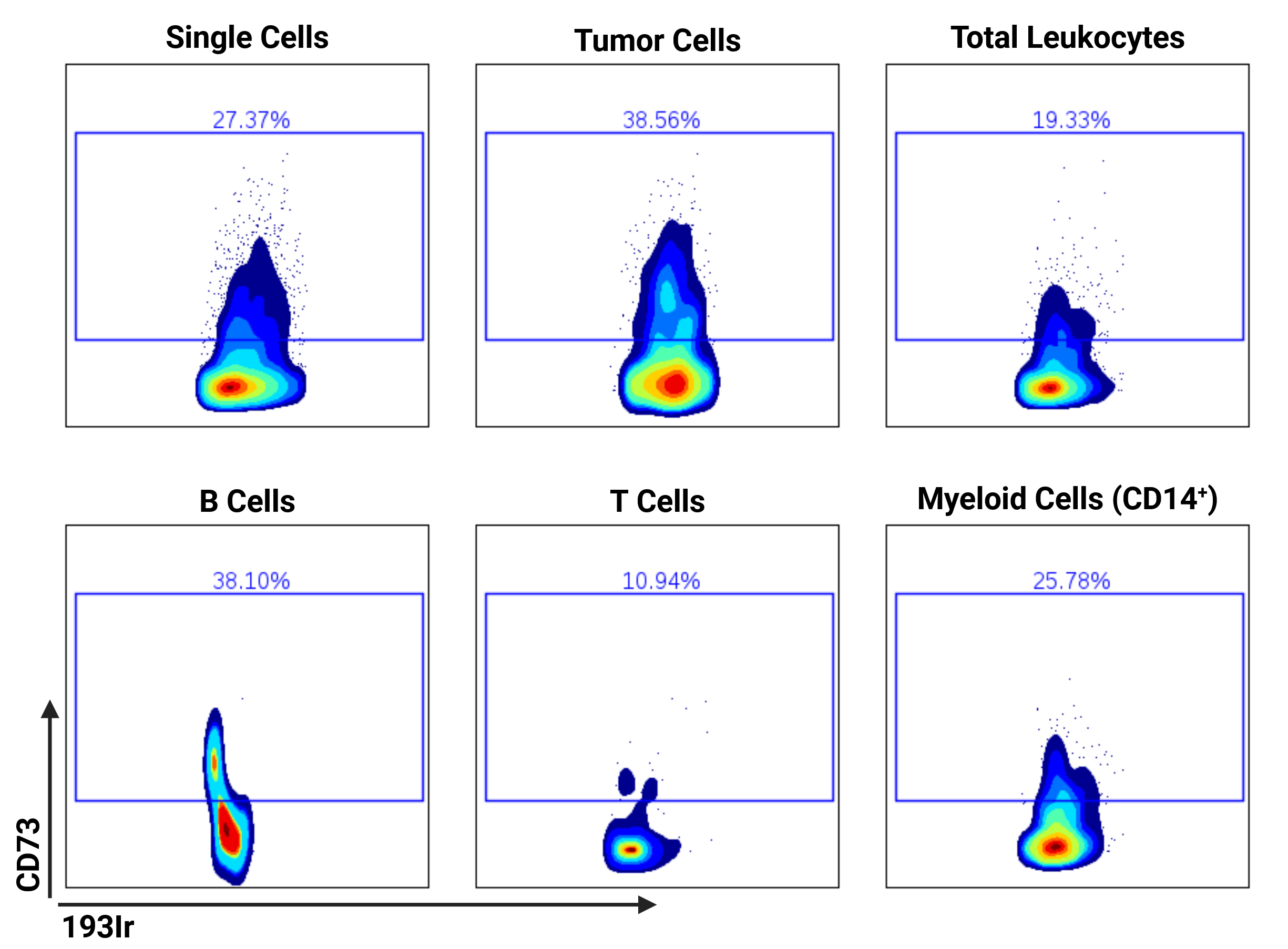


**Fig. S1** CD73 expression profiles of the constituents of the dissociated PDX material used in this study. Gates encompass cells above the CD73 positivity threshold, with the relative abundance of CD73-positive cells within the specified cell population displayed on top of each gate. The single-cell data used for this analysis was previously acquired using suspension mass cytometry.


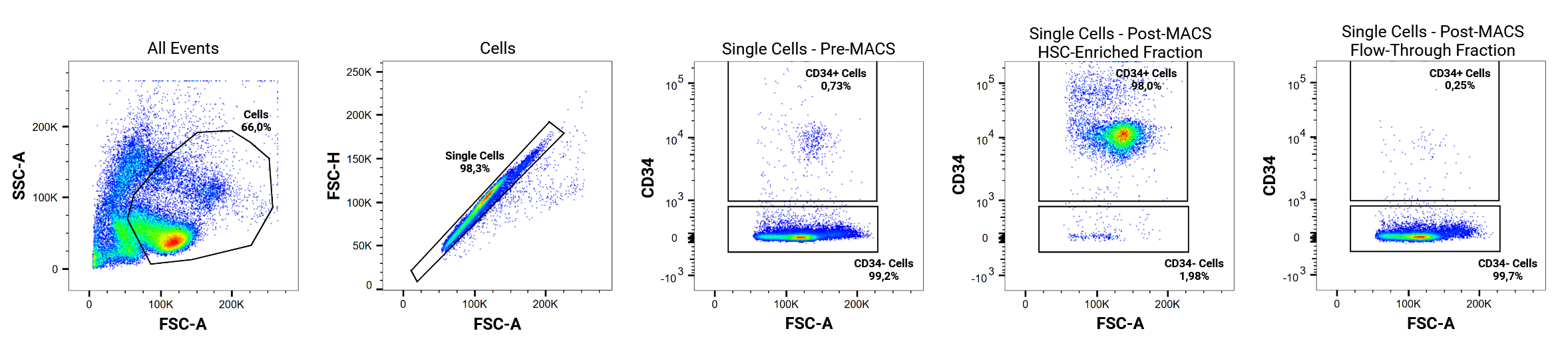


**Fig. S2** Gating strategy used for assessing the purity of samples enriched with human CD34^+^ hematopoietic stem cells from umbilical cord blood prior to their intravenous injection into NSGS mice. Enrichment was performed by magnetic activated cell sorting. Samples were analyzed using conventional flow cytometry. SSC - side scatter; FSC - forward scatter; A - area; H - height; HSC - hematopoietic stem cell; MACS - magnetic activated cell sorting


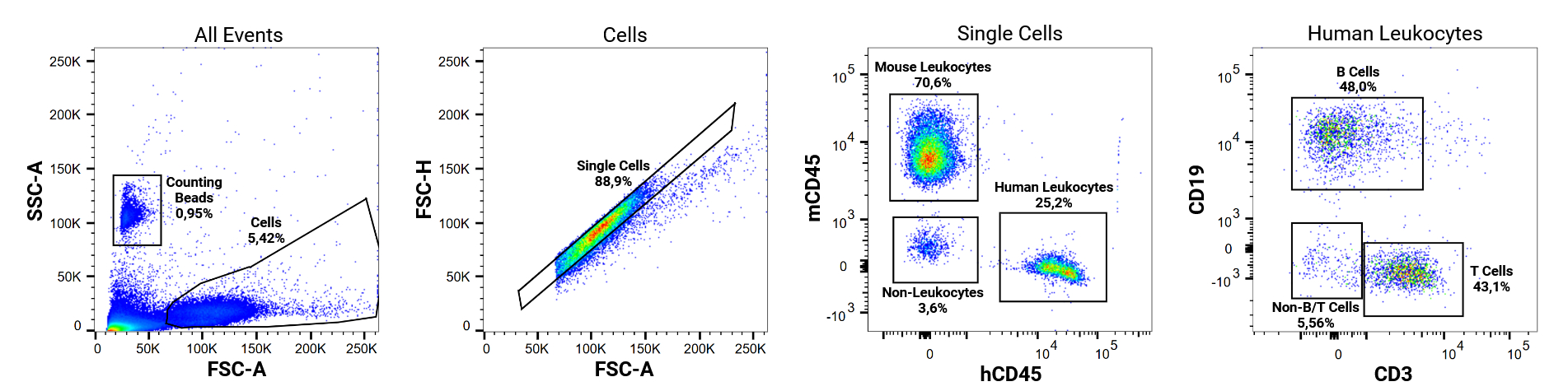


**Fig. S3** Representative gating strategy for the assessment of blood chimerism in mice injected with human hematopoietic stem cells. Leukocyte phenotyping was performed using fluorescence flow cytometry. Complete blood chimerism data is available in Table S4. SSC - side scatter; FSC - forward scatter; A - area; H - height; mCD45 - murine CD45; hCD45 - human CD45


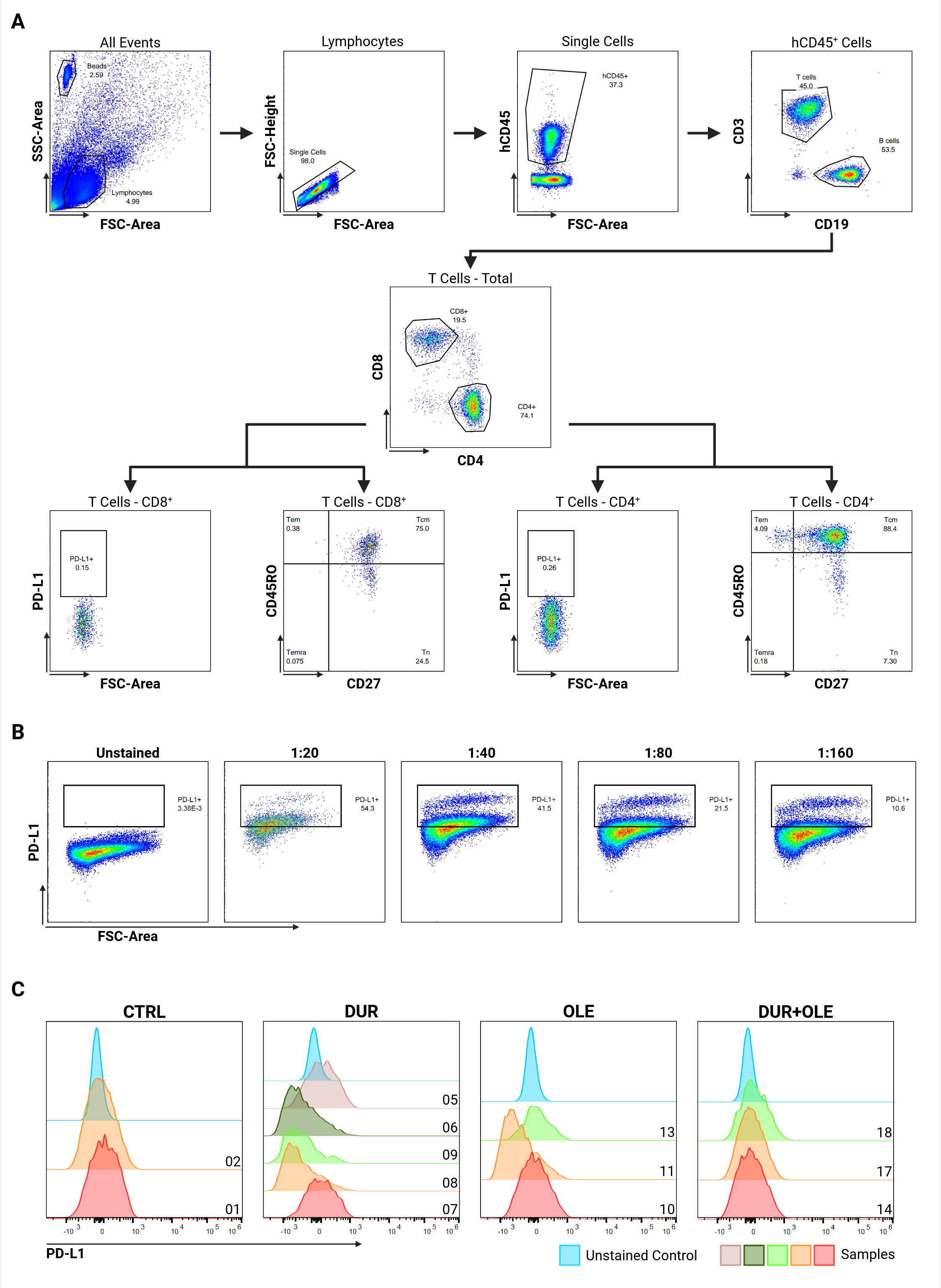


**Fig. S4** Key elements of the workflow used for the analysis of endpoint blood samples by spectral flow cytometry. (A) Titration of the anti-PD-L1 antibody. The sample used for titration consisted of peripheral blood mononuclear cells stimulated with phytohemagglutinin. The remainder of the spectral flow cytometry panel was titrated prior to this study (unpublished data). (B) Representative gating strategy used for the characterization of blood sample composition. (C) Histograms representing PD-L1 expression profiles of the total T-cell populations relative to the unstained control. Mouse IDs are displayed to the right of each histogram. Certain samples were excluded from this analysis due to low T-cell counts. Full PD-L1 expression data is available in Table S13. SSC - side scatter; FSC - forward scatter; Tn - naïve T cells; Tcm - central memory T cells; Tem - effector memory T cells; Temra - effector T cells; CTRL - control group; DUR - durvalumab-only group; OLE - oleclumab-only group; DUR+OLE - combination treatment group


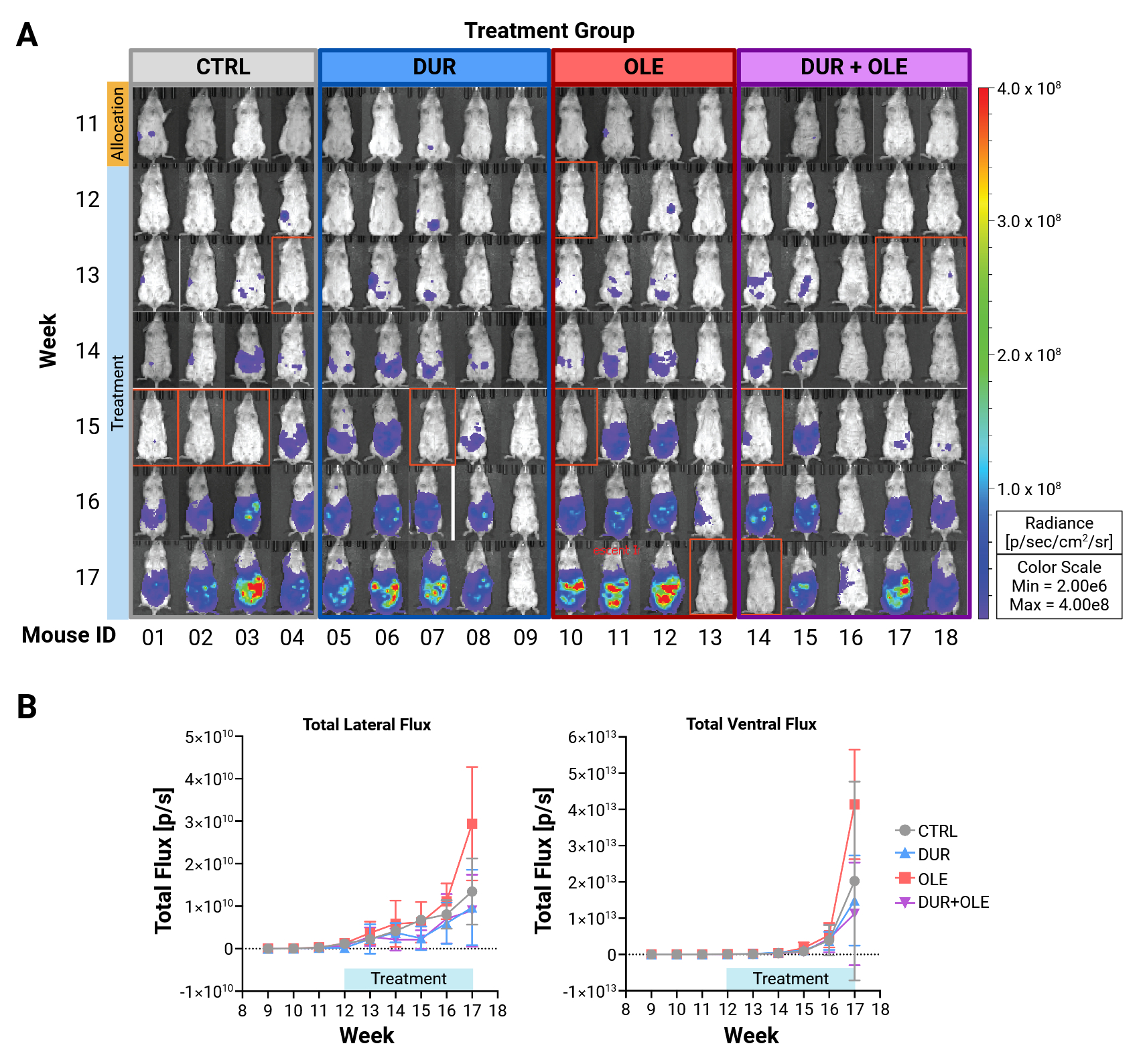


**Fig. S5** (A) Longitudinal overview of weekly bioluminescence imaging results for PDX-implanted experimental mice (n=18) in the ventral position. Each column represents an individual mouse. Only images from baseline (week 11) through endpoint (week 17) are shown. Mice outlined with an orange border displayed inexplicably low bioluminescence at the specified timepoint, even after luciferin re-injection. These data were excluded from further analyses. Full data on the total ventral flux are available in Table S5. (B) Average total absolute lateral (left graph) and ventral (right graph) photon flux in each treatment group during PDX engraftment evaluation (weeks 9-10), group allocation (week 11) and treatment (weeks 12-17). CTRL - control group; DUR - durvalumab-only group; OLE - oleclumab-only group; DUR+OLE - combination treatment group


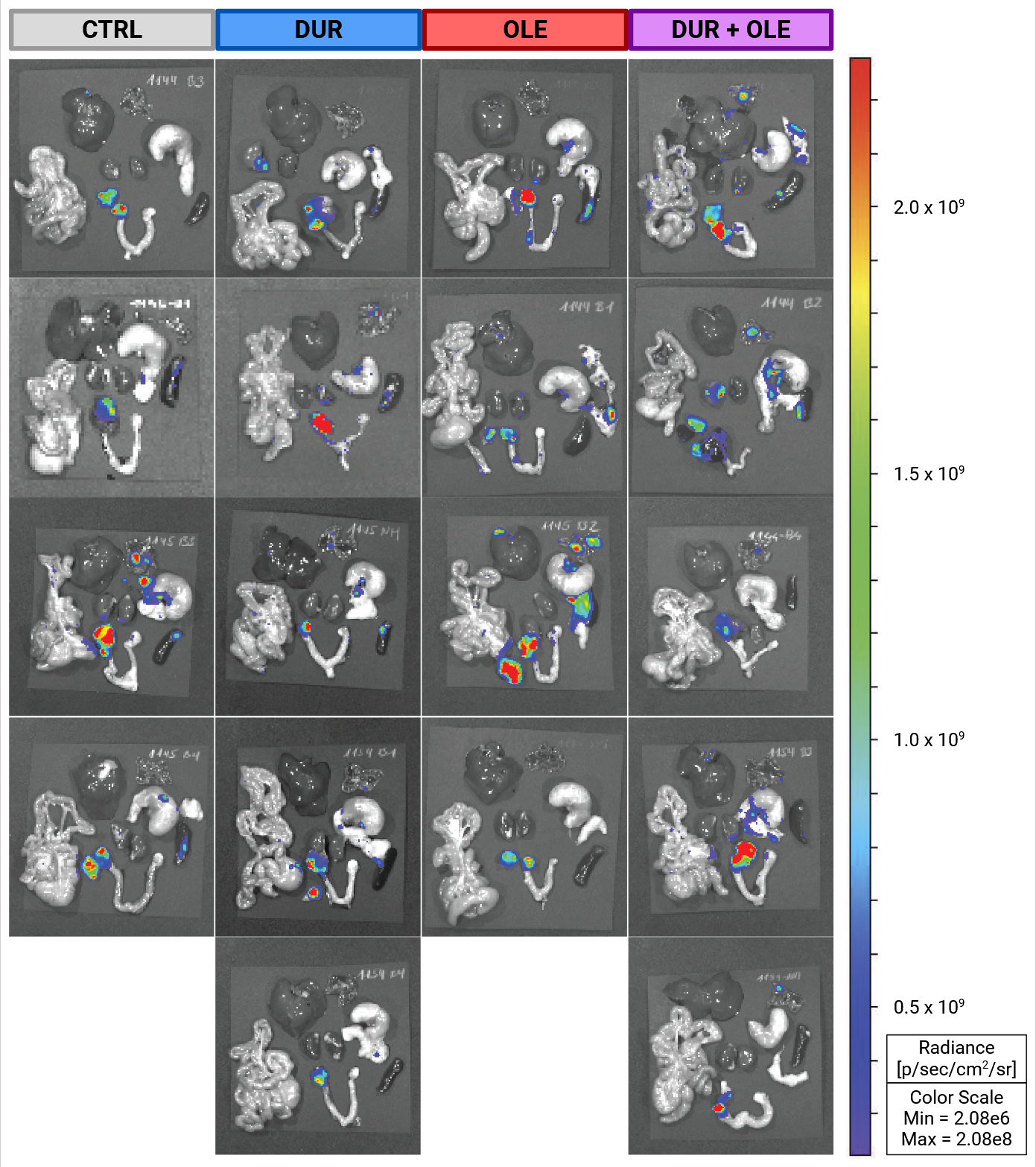


**Fig. S6** *Ex vivo* evaluation of intraabdominal tumor dissemination using BLI, presented for individual mice (n=18).

**
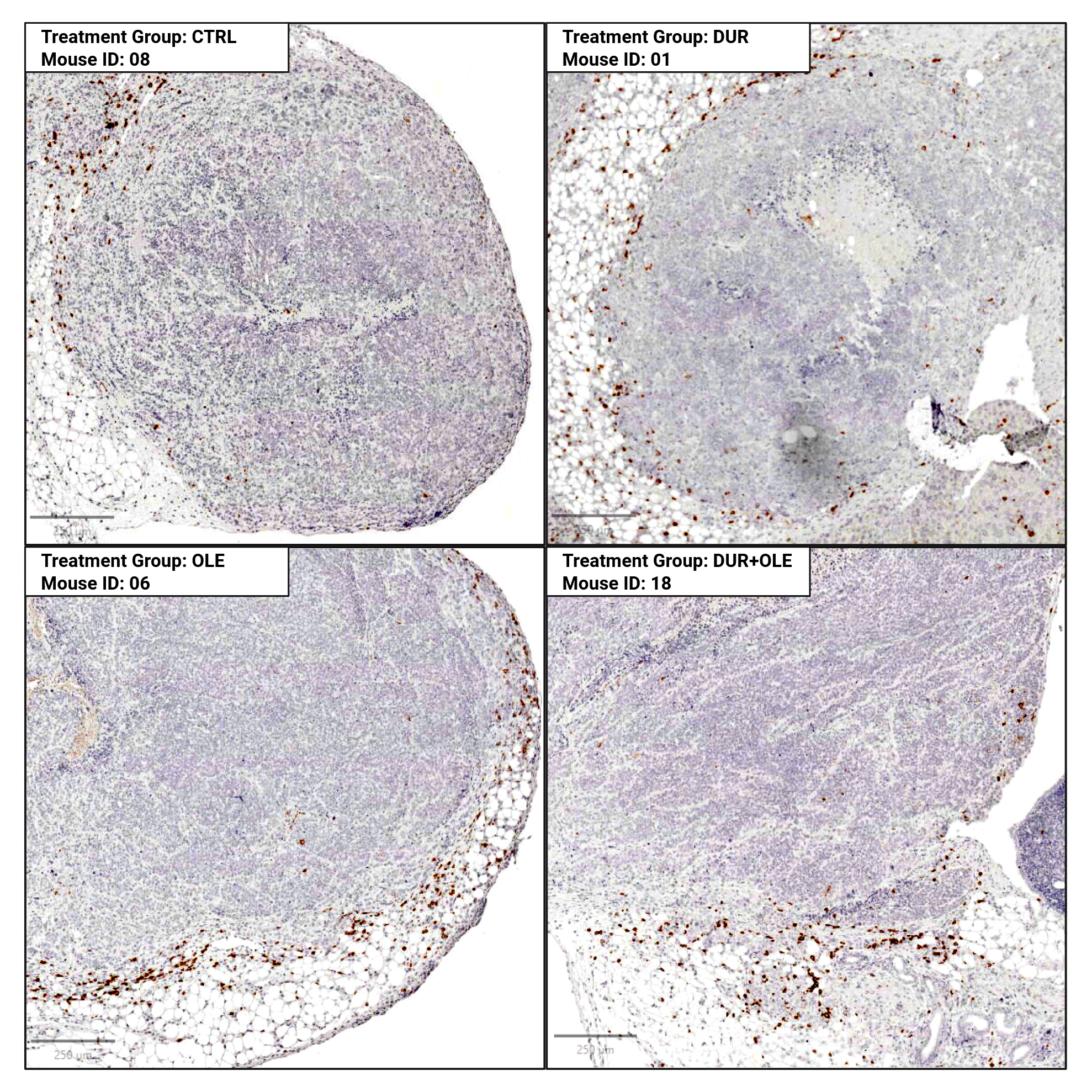
**

**Fig. S7** Light microscopy images (400x) of representative areas of the primary PDX tumor displaying prominent accumulation of human leukocytes in the invasive margin. Tumor sections were stained for human CD45. Each image encompasses a 2.5 mm^2^ area of a representative PDX tumor section from each treatment group (specified in the top left corner of each image). CTRL - control group; DUR - durvalumab-only group; OLE - oleclumab-only group; DUR+OLE - combination treatment group; PDX - patient-derived xenograft





**Fig. S8** Correlation plots showing associations between tumor burden at the end of the study and densities of intratumoral marker-positive leukocytes. Top row: all samples combined. Bottom four rows: samples from individual treatment groups. Significant correlations (p<0.05) are marked with an asterisk (*) in the graph title. Full correlation data is available in Table S9. Depending on the results of a Shapiro-Wilk normality test, either the Pearson or Spearman correlation test was used to determine the p-value for each dataset. The dashed lines represent the 95% confidence interval of the best-fit line. CTRL - control group; DUR - durvalumab-only group; OLE - oleclumab-only group; DUR+OLE - combination treatment group

**Table S1** Antibody panel used for the characterization of leukocytes in the blood samples from the experimental mice using spectral flow cytometry.

| **Fluorophore** | **Target** | **Clone** | **Dilution** | **Vendor** | **Cat.No.** | **Clonality** |
| --- | --- | --- | --- | --- | --- | --- |
| [BUV615](https://www.bdbiosciences.com/en-us/products/reagents/flow-cytometry-reagents/research-reagents/single-color-antibodies-ruo/buv615-mouse-anti-human-cd3.751157) | CD3 | HIT3a | 1:160 | BD | 751157 | Monoclonal |
| PerCP/Fire 806 | CD4 | SK3 | 1:160 | BioLegend | 344694 | Monoclonal |
| [Spark Blue 550](https://www.biolegend.com/en-us/products/spark-blue-550-anti-human-cd8-antibody-18494) | CD8 | SK1 | 1:160 | BioLegend | 344760 | Monoclonal |
| [PE/Fire 640](https://www.biolegend.com/en-us/products/pefire-640-anti-human-cd19-antibody-19533) | CD19 | HIB19 | 1:160 | BioLegend | 302274 | Monoclonal |
| [Spark Red 718](https://www.biolegend.com/en-us/products/spark-red-718-anti-human-cd27-recombinant-antibody-22289) | CD27 | QA17A18 | 1:80 | BioLegend | 393218 | Recombinant |
| [BV480](https://www.bdbiosciences.com/en-us/products/reagents/flow-cytometry-reagents/research-reagents/single-color-antibodies-ruo/bv480-mouse-anti-human-cd45ro.566143) | CD45RO | UCHL1 | 1:160 | BD | 566143 | Monoclonal |
| [BUV 805](https://www.thermofisher.com/antibody/product/368-0459-42.html?CID=AFLFF-368-0459-42) | CD45 | HI30 | 1:160 | ThermoFisher | 368-0459-42 | Monoclonal |
| [BV711](https://www.biolegend.com/en-gb/products/brilliant-violet-711-anti-human-cd274-b7-h1-pd-l1-antibody-11880?GroupID=BLG5404) | PD-L1 | 29E.2A3 | 1:80 | BioLegend | 329722 | Monoclonal |

BD - Becton, Dickinson and Company; BUV - Brilliant Ultra Violet; BV - Brilliant Violet

**Table S2** List of antibodies used for the immunohistochemical staining of primary patient-derived xenograft tumor sections.

| **Target** | **Clone** | **Host Species** | **Dilution** | **Vendor** | **Cat.No.** | **Clonality** |
| --- | --- | --- | --- | --- | --- | --- |
| hCD45 | 2B11 | Mouse | 1:100 | ThermoFisher | 14-9457-82 | Monoclonal |
| CD20 | H1 | Mouse | 1:200 | BD | 555677 | Monoclonal |
| CD3 | F7.2.38 | Mouse | 1:200 | Abcam | ab17143 | Monoclonal |
| CD8 | C8/144B | Mouse | 1:400 | BioLegend | 372902 | Monoclonal |
| FoxP3 | 236A/E7 | Mouse | 1:50 | ThermoFisher | 14-4777-82 | Monoclonal |

BD - Becton, Dickinson and Company; hCD45 - Human CD45

**Table S3** Overview of the positive cell detection parameters used for the enumeration of leukocytes in primary PDX tumor sections.

| **Parameter** | **hCD45, CD3, CD8** | **CD20** | **FoxP3** |
| --- | --- | --- | --- |
| Detection image | Optical density sum | | |
| Requested pixel size | 0.17 µm | | |
| Background Radius | 8 µm | | |
| Use opening by reconstruction | Yes | | |
| Median filter radius | 0 | | |
| Sigma | 1.5 | | |
| Minimum area | 20 | | |
| Maximum area | 201 | | |
| Threshold | 0.1 | | |
| Max background intensity | 2 | | |
| Split by shape | Yes | | |
| Exclude DAB (membrane staining) | No | | |
| Cell expansion | 3 µm | | |
| Include cell nucleus | Yes | | |
| Smooth boundaries | No | | |
| Make measurements | Yes | | |
| Score compartment | Cell: DAB OD Mean | Nucleus: DAB OD Mean | Nucleus: DAB OD Mean |
| Threshold 1+ | 0.04 | 0.2 | 0.3 |
| Threshold 2+ | 0.4 | | |
| Threshold 3+ | 0.6 | | |
| Single threshold | Yes | Yes | Yes |

**Table S4** Results of the chimerism assessments of mouse blood during model establishment and at the end of the study.

| **Mouse ID** | **Week 8**  **(PDX Implantation)** | | | **Week 11**  **(Group Assignment)** | | | **Week 17**  **(End of Study)** | | |
| --- | --- | --- | --- | --- | --- | --- | --- | --- | --- |
|  | **Human^a^** | **B Cells^b^** | **T Cells^b^** | **Human^a^** | **B Cells^b^** | **T Cells^b^** | **Human^a^** | **B Cells^b^** | **T Cells^b^** |
| 01 | 18.4 % | 90.7 % | N/A | 19.0 % | 88.8 % | 1.43 % | 21.9 % | 64.3 % | 28.8 % |
| 02 | 18.4 % | 88.5 % | N/A | 22.6 % | 85.3 % | 1.96 % | 51.6 % | 39.7 % | 51.3 % |
| 03 | 19.4 % | 89.1 % | N/A | 19.3 % | 88.4 % | 1.11 % | 24.0 % | 91.0 % | 0.75 % |
| 04 | 17.5 % | 86.6 % | N/A | 21.1 % | 86.4 % | 1.21 % | 28.2 % | 90.5 % | 0.96 % |
| 05 | 30.9 % | 87.4 % | N/A | 37.0 % | 88.8 % | 0.75 % | 22.6 % | 47.9 % | 42.7 % |
| 06 | 18.4 % | 86.6 % | N/A | 19.2 % | 82.4 % | 2.35 % | 29.1 % | 56.0 % | 31.7 % |
| 07 | 16.9 % | 86.5 % | N/A | 24.3 % | 86.9 % | 1.66 % | 20.0 % | 41.8 % | 48.1 % |
| 08 | 17.7 % | 87.0 % | N/A | 18.6 % | 56.2 % | 30.7 % | 22.2 % | 10.7 % | 77.3 % |
| 09 | 14.2 % | 87.1 % | N/A | 13.3 % | 86.8 % | 1.61 % | 11.9 % | 78.1 % | 14.9 % |
| 10 | 13.4 % | 85.6 % | N/A | 26.3 % | 88.3 % | 1.17 % | 28.7 % | 44.5 % | 41.6 % |
| 11 | 14.1 % | 90.8 % | N/A | 23.0 % | 88.8 % | 1.10 % | 29.4 % | 32.2 % | 57.4 % |
| 12 | 14.0 % | 88.2 % | N/A | 17.9 % | 87.0 % | 1.52 % | 25.8 % | 88.7 % | 0.68 % |
| 13 | 27.6 % | 88.2 % | N/A | 28.2 % | 87.0 % | 0.91 % | 29.8 % | 87.9 % | 1.52 % |
| 14 | 12.1 % | 75.0 % | N/A | 28.8 % | 89.6 % | 1.30 % | 39.4 % | 12.2 % | 79.6 % |
| 15 | 26.6 % | 88.9 % | N/A | 31.8 % | 87.5 % | 1.29 % | 20.0 % | 75.3 % | 2.12 % |
| 16 | 13.1 % | 92.6 % | N/A | 10.6 % | 90.9 % | 0.90 % | 8.14 % | 87.6 % | 1.42 % |
| 17 | 20.9 % | 90.4 % | N/A | 19.8 % | 80.3 % | 6.66 % | 30.4 % | 22.9 % | 70.6 % |
| 18 | 13.3 % | 88.6 % | N/A | 18.2 % | 86.8 % | 1.16 % | 14.2 % | 82.6 % | 9.52 % |

^a^ Percentage of single cells that are positive for human CD45.

^b^ Relative to total cells positive for human CD45.

**Table S5** Total lateral and ventral photon flux measured weekly during weeks 9 through 17 after injection of hematopoietic cells into the experimental mice.

| **Mouse ID** | **W09** | **W10** | **W11** | **W12** | **W13** | **W14** | **W15** | **W16** | **W17** |
| --- | --- | --- | --- | --- | --- | --- | --- | --- | --- |
| **Total Flux - Lateral** | | | | | | | | | |
| 01 | 1.49E+07 | 6.66E+07 | 2.67E+08 | 1.74E+09 | 2.43E+09 | 3.25E+09 | 5.00E+07 | 7.69E+09 | 5.98E+09 |
| 02 | 1.03E+07 | 2.74E+07 | 3.86E+08 | 7.00E+08 | 1.42E+09 | 3.38E+09 | 1.90E+06 | 3.49E+09 | 1.11E+10 |
| 03 | 3.93E+05 | 7.23E+07 | 2.67E+07 | 1.22E+09 | 2.87E+09 | 4.27E+09 | 3.76E+06 | 1.00E+10 | 2.44E+10 |
| 04 | 4.40E+05 | 4.84E+07 | 4.25E+08 | 1.32E+09 | 1.72E+06 | 5.85E+09 | 6.78E+09 | 1.11E+10 | 1.24E+10 |
| 05 | 4.39E+06 | 1.52E+07 | 8.25E+07 | 2.02E+08 | 1.40E+09 | 2.19E+09 | 3.30E+09 | 5.18E+09 | 6.60E+09 |
| 06 | 2.43E+07 | 9.86E+07 | 7.16E+08 | 2.92E+06 | 8.37E+09 | 6.81E+09 | 6.05E+09 | 1.29E+10 | 2.26E+10 |
| 07 | 4.43E+05 | 3.69E+07 | 2.36E+08 | 5.47E+08 | 1.48E+09 | 2.73E+09 | 4.21E+07 | 8.16E+09 | 1.45E+10 |
| 08 | 2.32E+06 | 8.93E+06 | 1.00E+08 | 8.16E+07 | 2.19E+08 | 1.53E+09 | 4.48E+08 | 3.84E+09 | 4.88E+09 |
| 09 | 7.80E+05 | 8.69E+05 | 2.30E+06 | 1.72E+06 | 3.82E+05 | 2.87E+07 | 5.17E+07 | 1.59E+06 | 2.95E+06 |
| 10 | 1.37E+06 | 6.96E+07 | 2.98E+08 | 2.93E+06 | 5.05E+09 | 5.81E+09 | 1.11E+08 | 1.10E+10 | 1.93E+10 |
| 11 | 1.36E+07 | 7.39E+07 | 2.35E+08 | 1.13E+09 | 2.65E+09 | 2.37E+09 | 4.00E+09 | 1.25E+10 | 2.44E+10 |
| 12 | 6.18E+05 | 1.63E+08 | 6.25E+08 | 2.42E+09 | 6.67E+09 | 1.36E+10 | 1.17E+10 | 1.56E+10 | 4.46E+10 |
| 13 | 4.43E+06 | 1.63E+07 | 4.49E+07 | 2.29E+08 | 5.03E+08 | 1.71E+09 | 2.92E+09 | 5.52E+09 | 1.26E+07 |
| 14 | 2.38E+07 | 6.75E+07 | 4.28E+08 | 2.03E+09 | 4.49E+09 | 5.52E+09 | 2.80E+07 | 1.43E+10 | 1.53E+07 |
| 15 | 7.69E+06 | 5.18E+07 | 3.39E+08 | 1.01E+09 | 2.69E+09 | 4.01E+09 | 5.42E+09 | 7.79E+09 | 7.86E+09 |
| 16 | 1.23E+06 | 6.33E+06 | 4.67E+07 | 7.20E+07 | 7.39E+08 | 7.30E+08 | 1.33E+09 | 5.80E+08 | 1.70E+09 |
| 17 | 5.89E+05 | 1.87E+07 | 2.22E+08 | 3.53E+08 | 6.41E+05 | 2.26E+07 | 5.51E+08 | 1.08E+10 | 2.11E+10 |
| 18 | 1.00E+06 | 1.68E+06 | 1.43E+07 | 4.19E+07 | 9.87E+05 | 2.22E+08 | 1.27E+09 | 1.90E+09 | 5.15E+09 |
| **Total Flux - Ventral** | | | | | | | | | |
| 01 | 2.01E+09 | 2.30E+09 | 1.13E+11 | 4.29E+10 | 8.02E+10 | 1.07E+11 | 5.55E+10 | 8.12E+11 | 2.69E+12 |
| 02 | 9.34E+08 | 7.25E+08 | 1.86E+09 | 1.70E+10 | 6.68E+10 | 1.39E+11 | 1.07E+09 | 1.79E+12 | 1.02E+13 |
| 03 | 4.78E+08 | 1.13E+09 | 1.31E+09 | 2.58E+10 | 2.13E+11 | 8.28E+11 | 2.02E+09 | 1.01E+13 | 6.11E+13 |
| 04 | 4.71E+08 | 2.74E+09 | 2.96E+09 | 3.30E+11 | 6.16E+09 | 2.27E+11 | 8.80E+11 | 2.97E+12 | 6.97E+12 |
| 05 | 8.04E+08 | 1.23E+09 | 3.48E+09 | 4.16E+09 | 6.82E+10 | 4.03E+11 | 1.49E+12 | 3.75E+12 | 1.26E+13 |
| 06 | 1.14E+09 | 1.41E+09 | 1.07E+10 | 8.71E+08 | 5.27E+11 | 1.42E+12 | 2.68E+12 | 6.89E+12 | 3.04E+13 |
| 07 | 5.22E+08 | 1.03E+10 | 4.50E+10 | 3.17E+11 | 2.26E+11 | 6.15E+11 | 1.91E+10 | 4.84E+12 | 2.43E+13 |
| 08 | 9.86E+08 | 1.28E+09 | 2.32E+09 | 9.14E+06 | 1.05E+10 | 2.06E+11 | 5.00E+11 | 3.31E+12 | 7.14E+12 |
| 09 | 1.08E+09 | 1.07E+09 | 1.15E+09 | 6.89E+08 | 1.00E+11 | 1.80E+09 | 2.96E+09 | 5.99E+08 | 6.62E+08 |
| 10 | 6.93E+08 | 1.65E+09 | 3.16E+09 | 5.98E+08 | 1.67E+11 | 2.41E+11 | 3.56E+10 | 4.99E+12 | 2.67E+13 |
| 11 | 1.34E+09 | 2.28E+09 | 8.80E+10 | 4.50E+10 | 2.44E+11 | 5.81E+11 | 2.63E+12 | 7.91E+12 | 4.05E+13 |
| 12 | 5.85E+08 | 6.96E+09 | 2.80E+10 | 2.35E+11 | 2.83E+11 | 6.34E+11 | 2.73E+12 | 7.68E+12 | 5.69E+13 |
| 13 | 1.49E+09 | 1.59E+09 | 2.02E+09 | 6.24E+09 | 3.38E+09 | 4.48E+10 | 2.60E+10 | 6.71E+11 | 1.27E+09 |
| 14 | 9.20E+08 | 2.17E+09 | 1.31E+10 | 5.20E+10 | 3.13E+11 | 7.87E+11 | 2.04E+11 | 9.18E+12 | 6.15E+09 |
| 15 | 1.54E+09 | 5.09E+09 | 1.90E+10 | 8.57E+10 | 2.88E+11 | 4.92E+11 | 2.63E+12 | 6.89E+12 | 8.13E+12 |
| 16 | 1.07E+09 | 8.63E+08 | 1.86E+09 | 6.00E+09 | 2.66E+10 | 1.57E+10 | 7.75E+10 | 6.85E+10 | 3.30E+11 |
| 17 | 6.70E+08 | 2.98E+09 | 1.81E+09 | 9.92E+09 | 3.73E+08 | 5.62E+09 | 1.94E+11 | 4.68E+12 | 3.19E+13 |
| 18 | 1.04E+09 | 9.25E+08 | 1.65E+09 | 1.44E+09 | 5.75E+08 | 1.87E+10 | 1.57E+11 | 1.15E+12 | 4.52E+12 |

W - week

**Table S6** Dimensions of primary patient-derived xenograft tumors measured at the end of the study. Tumor volumes were calculated using the formula: (height x width x length x π) / 6.

| **Mouse ID** | **Tumor Dimensions** | | | | |
| --- | --- | --- | --- | --- | --- |
|  | **Height [mm]** | **Width [mm]** | **Length [mm]** | **Volume [mm^3^]** | **Weight [mg]** |
| 01 | 2.8 | 3.4 | 5.32 | 26.52 | 85 |
| 02 | 8.35 | 3.32 | 13.24 | 192.18 | 580 |
| 03 | 5.73 | 8.38 | 9.65 | 242.62 | 280 |
| 04 | 6.47 | 6.1 | 7.88 | 162.84 | 141 |
| 05 | 11.27 | 7.45 | 10.73 | 471.71 | 848 |
| 06 | 4.99 | 6.14 | 10.61 | 170.21 | 189 |
| 07 | 4.34 | 3.26 | 6.72 | 49.78 | 56 |
| 08 | 5.8 | 9.39 | 8.43 | 240.39 | 266 |
| 09 | 4.2 | 5.04 | 6.57 | 72.82 | 100 |
| 10 | 6.92 | 5.51 | 4.07 | 81.26 | 102 |
| 11 | 5.03 | 6.54 | 5.33 | 91.81 | 103 |
| 12 | 5.43 | 7.66 | 6.88 | 149.84 | 205 |
| 13 | 5.17 | 4.86 | 3.59 | 47.23 | 55 |
| 14 | 7.4 | 4.91 | 8.04 | 152.96 | 102 |
| 15 | 10.09 | 10.72 | 14.11 | 799.12 | 964 |
| 16 | 10.75 | 7.02 | 7.13 | 281.73 | 246 |
| 17 | 6.2 | 10.8 | 5.95 | 208.61 | 253 |
| 18^a^ | - | - | - | - | - |

^a^ Primary tumor too small to measure.

**Table S7** The extent of visible metastatic dissemination at the end of the study.

| **Mouse ID** | **Anatomic Localization of Visible Metastatic Lesion(s)** | | | | | | | | |
| --- | --- | --- | --- | --- | --- | --- | --- | --- | --- |
|  | **DIA** | **DUO** | **GAS** | **HEP** | **MES** | **OME** | **PER** | **REN** | **SPL** |
| 01 |  |  |  | x |  |  | x |  | x |
| 02 |  |  |  | x |  |  | x |  |  |
| 03 | x |  | x |  |  |  |  |  |  |
| 04 |  |  |  |  |  | x | x |  |  |
| 05 |  |  |  |  | x | x |  | x |  |
| 06 | x |  |  |  |  |  | x |  |  |
| 07 | x |  |  |  |  |  | x | x | x |
| 08 | x | x |  |  |  | x | x |  |  |
| 09 |  |  |  |  |  |  |  |  |  |
| 10 |  |  |  |  |  | x | x |  |  |
| 11 |  |  |  |  |  |  | x |  |  |
| 12 | x |  |  | x |  | x | x |  |  |
| 13 |  |  |  |  |  |  | x |  |  |
| 14 | x |  |  |  |  | x | x |  |  |
| 15 | x |  | x |  |  | x | x | x | x |
| 16 | x |  |  |  |  |  |  |  |  |
| 17 |  |  |  |  |  |  |  |  |  |
| 18 | x |  |  |  |  |  |  |  |  |

DIA - diaphragmatic; DUO - duodenal; GAS - gastric; HEP - hepatic; MES - mesenteric; OME - omental; PER - peritoneal wall; REN - renal; SPL - splenic

**Table S8** Results of the digital analysis of primary patient-derived xenograft tumor sections. The total size of the annotated area is expressed as mm^2^, and the positive cell density is expressed as the total number of positive cells per mm^2^ of the total annotated area.

| **Mouse ID** | **Parameter** | **Marker** | | | | |
| --- | --- | --- | --- | --- | --- | --- |
|  |  | **hCD45** | **CD20** | **CD3** | **CD8** | **FoxP3** |
| 01 | Area | 0.65 | 0.65 | 0.65 | 0.51 | 0.65 |
|  | Count | 226 | 97 | 171 | 16 | 15 |
|  | Density | 346.4 | 148.7 | 262.1 | 31.1 | 23.2 |
| 02 | Area | 1.99 | 1.99 | 1.97 | 1.99 | 1.95 |
|  | Count | 1094 | 424 | 1037 | 324 | 104 |
|  | Density | 548.5 | 212.6 | 526.1 | 162.4 | 53.3 |
| 03 | Area | 0.81 | 0.80 | 0.81 | 0.76 | 0.78 |
|  | Count | 58 | 30 | 15 | 13 | 5 |
|  | Density | 71.2 | 37.6 | 18.4 | 17.1 | 6.4 |
| 04 | Area | 1.60 | 1.60 | 1.60 | 1.50 | 1.60 |
|  | Count | 215 | 221 | 6 | 10 | 3 |
|  | Density | 134.4 | 138.2 | 3.8 | 6.7 | 1.9 |
| 05 | Area | 1.47 | 1.46 | 1.47 | 1.47 | 1.47 |
|  | Count | 459 | 230 | 338 | 141 | 136 |
|  | Density | 311.3 | 157.5 | 229.2 | 95.6 | 92.7 |
| 06 | Area | 1.38 | 1.38 | 1.37 | 1.38 | 1.37 |
|  | Count | 316 | 74 | 164 | 56 | 9 |
|  | Density | 228.8 | 53.8 | 119.6 | 40.5 | 6.5 |
| 07^a^ | - | - | - | - | - | - |
| 08 | Area | 0.79 | 0.79 | 0.79 | 0.76 | 0.79 |
|  | Count | 116 | 19 | 141 | 56 | 19 |
|  | Density | 146.0 | 23.9 | 177.5 | 73.4 | 23.9 |
| 09 | Area | 0.90 | 0.90 | 0.80 | 0.90 | 0.90 |
|  | Count | 37 | 10 | 43 | 22 | 6 |
|  | Density | 40.9 | 11.1 | 53.4 | 24.3 | 6.7 |
| 10 | Area | 0.91 | 0.93 | 0.93 | 0.93 | 0.93 |
|  | Count | 110 | 35 | 79 | 18 | 14 |
|  | Density | 121.1 | 37.5 | 84.6 | 19.3 | 15.0 |
| 11 | Area | 1.34 | 1.29 | 1.33 | 1.34 | 1.34 |
|  | Count | 330 | 41 | 174 | 30 | 10 |
|  | Density | 247.0 | 31.8 | 130.6 | 22.5 | 7.5 |
| 12 | Area | 1.00 | 1.00 | 1.00 | 0.99 | 0.98 |
|  | Count | 68 | 47 | 9 | 15 | 4 |
|  | Density | 67.8 | 46.8 | 9.0 | 15.1 | 4.1 |
| 13 | Area | 0.86 | 0.86 | 0.85 | 0.86 | 0.86 |
|  | Count | 66 | 54 | 14 | 15 | 2 |
|  | Density | 76.7 | 62.8 | 16.5 | 17.4 | 2.3 |
| 14 | Area | 0.69 | 0.69 | 0.69 | 0.69 | 0.69 |
|  | Count | 362 | 34 | 341 | 83 | 50 |
|  | Density | 521.6 | 49.0 | 491.3 | 119.6 | 72.0 |
| 15 | Area | 1.15 | 1.15 | 0.76 | 1.06 | 1.06 |
|  | Count | 782 | 399 | 7 | 12 | 1 |
|  | Density | 679.0 | 346.5 | 9.2 | 11.3 | 0.9 |
| 16 | Area | 0.72 | 0.72 | 0.72 | 0.72 | 0.72 |
|  | Count | 174 | 238 | 1 | 3 | 22 |
|  | Density | 241.9 | 330.8 | 1.4 | 4.2 | 30.6 |
| 17 | Area | 2.53 | 2.52 | 2.53 | 2.53 | 2.43 |
|  | Count | 1034 | 69 | 795 | 411 | 51 |
|  | Density | 409.4 | 27.4 | 314.8 | 162.7 | 21.0 |
| 18 | Area | 0.86 | 0.82 | 0.86 | 0.83 | 0.86 |
|  | Count | 34 | 22 | 38 | 49 | 9 |
|  | Density | 39.4 | 26.7 | 44.0 | 59.3 | 10.4 |

^a^ No discernable invasive tumor margin present.

**Table S9** Parameters of the correlations between tumor burden at the end of the study and densities of intratumoral marker-positive leukocytes for all samples combined and each treatment group individually.

| **Treatment**  **Group** | **Parameter** | **Pearson’s Correlation Coefficient (r)**  **Goodness of Fit (R^2^)**  **p-value** | | | | |
| --- | --- | --- | --- | --- | --- | --- |
|  |  | **hCD45** | **CD20** | **CD3** | **CD8** | **FoxP3** |
| All  Samples | r^a^ | 0.382 | 0.359 | -0.082 | -0.029 | 0.159 |
|  | R^2^ | 0.317 | 0.433 | 0.016 | 0.001 | 0.029 |
|  | p | 0.145 | 0.173 | 0.763 | 0.917 | 0.556 |
| Control | r | -0.299 | -0.368 | -0.195 | 0.167 | -0.079 |
|  | R^2^ | 0.089 | 0.135 | 0.038 | 0.028 | 0.006 |
|  | p | 0.701 | 0.632 | 0.805 | 0.833 | 0.921 |
| Durvalumab | r | 0.853 | 0.922 | 0.950 | 0.952 | 0.961 |
|  | R^2^ | 0.728 | 0.850 | 0.902 | 0.907 | 0.924 |
|  | p | 0.147 | 0.078 | 0.050 | 0.048 | 0.039 |
| Oleclumab | r | -0.107 | -0.351 | -0.173 | -0.392 | -0.061 |
|  | R^2^ | 0.012 | 0.123 | 0.030 | 0.153 | 0.004 |
|  | p | 0.893 | 0.649 | 0.828 | 0.608 | 0.939 |
| Durvalumab  +  Oleclumab | r | 0.659 | 0.720 | -0.680 | -0.638 | -0.756 |
|  | R^2^ | 0.434 | 0.518 | 0.463 | 0.407 | 0.571 |
|  | p | 0.341 | 0.280 | 0.320 | 0.362 | 0.244 |

r - Pearson’s correlation coefficient

R^2^ - goodness of fit parameter of the results of simple linear regression

p - p-value

^a^ Spearman’s rank correlation used due to non-normality of dataset.

**Table S10** Results of the spectral flow cytometry analysis of leukocytes in the blood samples from the experimental mice - human leukocyte counts.

| **Mouse ID** | **Total**  **Human**  **Leukocytes** | **T Cells** | | | | | | | | | | |
| --- | --- | --- | --- | --- | --- | --- | --- | --- | --- | --- | --- | --- |
|  |  | **Total** | **CD4+** | | | | | **CD8+** | | | | |
|  |  |  | **Total** | **N** | **CM** | **EM** | **E** | **Total** | **N** | **CM** | **EM** | **E** |
| 01 | 11758 | 2687 | 2281 | 115 | 1976 | 188 | 2 | 317 | 80 | 237 | 0 | 0 |
| 02 | 92523 | 47690 | 42615 | 997 | 29170 | 12211 | 237 | 4197 | 472 | 3720 | 5 | 0 |
| 03 | 20376 | 24 | 2 | 1 | 0 | 1 | 0 | 8 | 7 | 1 | 0 | 0 |
| 04 | 11458 | 18 | 0 | 0 | 0 | 0 | 0 | 1 | 1 | 0 | 0 | 0 |
| 05 | 15147 | 6823 | 5057 | 369 | 4472 | 207 | 9 | 1333 | 327 | 1000 | 5 | 1 |
| 06 | 27069 | 7650 | 4705 | 214 | 3300 | 1169 | 22 | 1583 | 154 | 1400 | 25 | 4 |
| 07 | 4735 | 2028 | 1948 | 95 | 1519 | 330 | 4 | 50 | 9 | 40 | 0 | 1 |
| 08 | 4726 | 3856 | 3344 | 30 | 2731 | 571 | 12 | 313 | 36 | 268 | 7 | 2 |
| 09 | 4616 | 345 | 257 | 67 | 121 | 65 | 4 | 48 | 27 | 21 | 0 | 0 |
| 10 | 14045 | 5025 | 4216 | 113 | 3035 | 1057 | 11 | 453 | 44 | 408 | 1 | 0 |
| 11 | 23690 | 14465 | 12695 | 252 | 10262 | 2157 | 24 | 1281 | 207 | 1069 | 3 | 2 |
| 12 | 25128 | 28 | 0 | 0 | 0 | 0 | 0 | 3 | 3 | 0 | 0 | 0 |
| 13 | 23542 | 109 | 48 | 15 | 30 | 3 | 0 | 32 | 26 | 4 | 0 | 2 |
| 14 | 16152 | 13406 | 11552 | 334 | 8703 | 2391 | 124 | 731 | 82 | 649 | 0 | 0 |
| 15 | 11661 | 28 | 14 | 4 | 10 | 0 | 0 | 6 | 5 | 1 | 0 | 0 |
| 16 | 9711 | 2 | 0 | 0 | 0 | 0 | 0 | 0 | 0 | 0 | 0 | 0 |
| 17 | 47863 | 35237 | 26539 | 1130 | 22368 | 2821 | 220 | 6140 | 297 | 5805 | 27 | 11 |
| 18 | 15658 | 1145 | 654 | 63 | 332 | 245 | 14 | 249 | 141 | 105 | 2 | 1 |

CM - Central Memory; E - Effector (Temra); EM - Effector Memory; N - Naïve

**Table S11** Results of the spectral flow cytometry analysis of leukocytes in the blood samples from the experimental mice - human leukocyte counts per µL of blood.

| **Mouse ID** | **Total**  **Human**  **Leukocytes** | **T Cells** | | | | | | | | | | |
| --- | --- | --- | --- | --- | --- | --- | --- | --- | --- | --- | --- | --- |
|  |  | **Total** | **CD4+** | | | | | **CD8+** | | | | |
|  |  |  | **Total** | **N** | **CM** | **EM** | **E** | **Total** | **N** | **CM** | **EM** | **E** |
| 01 | 91.6 | 20.9 | 17.8 | 0.9 | 15.4 | 1.5 | 0.0 | 2.5 | 0.6 | 1.8 | 0.0 | 0.0 |
| 02 | 608.6 | 313.7 | 280.3 | 6.6 | 191.9 | 80.3 | 1.6 | 27.6 | 3.1 | 24.5 | 0.0 | 0.0 |
| 03 | 168.3 | 0.2 | 0.0 | 0.0 | 0.0 | 0.0 | 0.0 | 0.1 | 0.1 | 0.0 | 0.0 | 0.0 |
| 04 | 186.8 | 0.3 | 0.0 | 0.0 | 0.0 | 0.0 | 0.0 | 0.0 | 0.0 | 0.0 | 0.0 | 0.0 |
| 05 | 140.5 | 63.3 | 46.9 | 3.4 | 41.5 | 1.9 | 0.1 | 12.4 | 3.0 | 9.3 | 0.0 | 0.0 |
| 06 | 377.2 | 106.6 | 65.6 | 3.0 | 46.0 | 16.3 | 0.3 | 22.1 | 2.1 | 19.5 | 0.3 | 0.1 |
| 07 | 38.7 | 16.6 | 15.9 | 0.8 | 12.4 | 2.7 | 0.0 | 0.4 | 0.1 | 0.3 | 0.0 | 0.0 |
| 08 | 73.5 | 59.9 | 52.0 | 0.5 | 42.5 | 8.9 | 0.2 | 4.9 | 0.6 | 4.2 | 0.1 | 0.0 |
| 09 | 56.5 | 4.2 | 3.1 | 0.8 | 1.5 | 0.8 | 0.0 | 0.6 | 0.3 | 0.3 | 0.0 | 0.0 |
| 10 | 203.0 | 72.6 | 60.9 | 1.6 | 43.9 | 15.3 | 0.2 | 6.5 | 0.6 | 5.9 | 0.0 | 0.0 |
| 11 | 369.9 | 225.9 | 198.2 | 3.9 | 160.2 | 33.7 | 0.4 | 20.0 | 3.2 | 16.7 | 0.0 | 0.0 |
| 12 | 228.6 | 0.3 | 0.0 | 0.0 | 0.0 | 0.0 | 0.0 | 0.0 | 0.0 | 0.0 | 0.0 | 0.0 |
| 13 | 185.9 | 0.9 | 0.4 | 0.1 | 0.2 | 0.0 | 0.0 | 0.3 | 0.2 | 0.0 | 0.0 | 0.0 |
| 14 | 157.6 | 130.8 | 112.7 | 3.3 | 84.9 | 23.3 | 1.2 | 7.1 | 0.8 | 6.3 | 0.0 | 0.0 |
| 15 | 215.3 | 0.5 | 0.3 | 0.1 | 0.2 | 0.0 | 0.0 | 0.1 | 0.1 | 0.0 | 0.0 | 0.0 |
| 16 | 93.5 | 0.0 | 0.0 | 0.0 | 0.0 | 0.0 | 0.0 | 0.0 | 0.0 | 0.0 | 0.0 | 0.0 |
| 17 | 420.9 | 309.8 | 233.4 | 9.9 | 196.7 | 24.8 | 1.9 | 54.0 | 2.6 | 51.0 | 0.2 | 0.1 |
| 18 | 107.1 | 7.8 | 4.5 | 0.4 | 2.3 | 1.7 | 0.1 | 1.7 | 1.0 | 0.7 | 0.0 | 0.0 |

CM - Central Memory; E - Effector (Temra); EM - Effector Memory; N - Naive

**Table S12** Results of the spectral flow cytometry analysis of leukocytes in the blood samples from the experimental mice - human leukocyte abundances relative to total human leukocytes. Values in the cells are expressed as percentages.

| **Mouse ID** | **Total**  **Human**  **Leukocytes** | **T Cells** | | | | | | | | | | |
| --- | --- | --- | --- | --- | --- | --- | --- | --- | --- | --- | --- | --- |
|  |  | **Total** | **CD4+** | | | | | **CD8+** | | | | |
|  |  |  | **Total** | **N** | **CM** | **EM** | **E** | **Total** | **N** | **CM** | **EM** | **E** |
| 01 | 100.0 | 22.9 | 19.4 | 1.0 | 16.8 | 1.6 | 0.0 | 2.7 | 0.7 | 2.0 | 0.0 | 0.0 |
| 02 | 100.0 | 51.5 | 46.1 | 1.1 | 31.5 | 13.2 | 0.3 | 4.5 | 0.5 | 4.0 | 0.0 | 0.0 |
| 03 | 100.0 | 0.1 | 0.0 | 0.0 | 0.0 | 0.0 | 0.0 | 0.0 | 0.0 | 0.0 | 0.0 | 0.0 |
| 04 | 100.0 | 0.2 | 0.0 | 0.0 | 0.0 | 0.0 | 0.0 | 0.0 | 0.0 | 0.0 | 0.0 | 0.0 |
| 05 | 100.0 | 45.0 | 33.4 | 2.4 | 29.5 | 1.4 | 0.1 | 8.8 | 2.2 | 6.6 | 0.0 | 0.0 |
| 06 | 100.0 | 28.3 | 17.4 | 0.8 | 12.2 | 4.3 | 0.1 | 5.8 | 0.6 | 5.2 | 0.1 | 0.0 |
| 07 | 100.0 | 42.8 | 41.1 | 2.0 | 32.1 | 7.0 | 0.1 | 1.1 | 0.2 | 0.8 | 0.0 | 0.0 |
| 08 | 100.0 | 81.6 | 70.8 | 0.6 | 57.8 | 12.1 | 0.3 | 6.6 | 0.8 | 5.7 | 0.1 | 0.0 |
| 09 | 100.0 | 7.5 | 5.6 | 1.5 | 2.6 | 1.4 | 0.1 | 1.0 | 0.6 | 0.5 | 0.0 | 0.0 |
| 10 | 100.0 | 35.8 | 30.0 | 0.8 | 21.6 | 7.5 | 0.1 | 3.2 | 0.3 | 2.9 | 0.0 | 0.0 |
| 11 | 100.0 | 61.1 | 53.6 | 1.1 | 43.3 | 9.1 | 0.1 | 5.4 | 0.9 | 4.5 | 0.0 | 0.0 |
| 12 | 100.0 | 0.1 | 0.0 | 0.0 | 0.0 | 0.0 | 0.0 | 0.0 | 0.0 | 0.0 | 0.0 | 0.0 |
| 13 | 100.0 | 0.5 | 0.2 | 0.1 | 0.1 | 0.0 | 0.0 | 0.1 | 0.1 | 0.0 | 0.0 | 0.0 |
| 14 | 100.0 | 83.0 | 71.5 | 2.1 | 53.9 | 14.8 | 0.8 | 4.5 | 0.5 | 4.0 | 0.0 | 0.0 |
| 15 | 100.0 | 0.2 | 0.1 | 0.0 | 0.1 | 0.0 | 0.0 | 0.1 | 0.0 | 0.0 | 0.0 | 0.0 |
| 16 | 100.0 | 0.0 | 0.0 | 0.0 | 0.0 | 0.0 | 0.0 | 0.0 | 0.0 | 0.0 | 0.0 | 0.0 |
| 17 | 100.0 | 73.6 | 55.4 | 2.4 | 46.7 | 5.9 | 0.5 | 12.8 | 0.6 | 12.1 | 0.1 | 0.0 |
| 18 | 100.0 | 7.3 | 4.2 | 0.4 | 2.1 | 1.6 | 0.1 | 1.6 | 0.9 | 0.7 | 0.0 | 0.0 |

CM - Central Memory; E - Effector (Temra); EM - Effector Memory; N - Naïve

**Table S13** PD-L1 expression levels on T cells and relative abundances of PD-L1-positive T cells in mouse blood taken at the end of the study.

| **Mouse ID** | **PD-L1 MFI**  **T Cells^a^** | **PD-L1 MFI**  **CD4^+^ Cells^a^** | **%PD-L1^+^**  **CD4^+^ Cells^b^** | **PD-L1 MFI**  **CD8^+^ Cells^a^** | **%PD-L1^+^**  **CD8^+^ Cells^b^** |
| --- | --- | --- | --- | --- | --- |
| 01 | 118 | 117 | 0,088 | 146 | 0,32 |
| 02 | 35,8 | 33,1 | 0,12 | 53,7 | 0,17 |
| 03^c^ | 19,7 | -70,8 | 0 | -7,15 | 0 |
| 04^c^ | -427 | N/A | 0 | -267 | 0 |
| 05 | 125 | 142 | 0,26 | 80,7 | 0,15 |
| 06 | -369 | -352 | 0,28 | -417 | 0,13 |
| 07 | 102 | 98,8 | 0,15 | 189 | 0 |
| 08 | -436 | -437 | 0,06 | -456 | 0 |
| 09 | -382 | -350 | 0 | -441 | 0 |
| 10 | 23,2 | 16,1 | 0,17 | 78,9 | 0,44 |
| 11 | -368 | -370 | 0,047 | -366 | 0,078 |
| 12^c^ | 54,6 | N/A | 0 | -53,7 | 0 |
| 13 | 71,7 | 140 | 0 | -29,5 | 0 |
| 14 | 15,2 | 8,94 | 0,061 | 115 | 0,14 |
| 15^c^ | -287 | -263 | 0 | -799 | 0 |
| 16^c^ | 183 | N/A | 0 | N/A | 0 |
| 17 | 19,7 | 20,6 | 0,064 | 8,04 | 0,016 |
| 18 | 78 | 108 | 0,46 | 29,5 | 0 |

MFI - median fluorescence intensity

^a^ Expressed as median fluorescence intensity relative to an unstained control sample.

^b^ Abundance is relative to total CD4^+^ or CD8^+^ T-cell population.

^c^ Sample contained insufficient T-cells for measuring PD-L1 expression.

**Supplementary Protocol 1: Isolation of Hematopoietic Stem Cells from Umbilical Cord Blood**

Ensure that the working environment and solutions used in this procedure are clean and sterile.

Solutions should be acclimated to room temperature (RT), unless specified otherwise in the protocol.

While the choice of density gradient medium is left up to the reader, this protocol is based on the usage of Lymphoprep™ (Cat.No. 07861, Stemcell Technologies, Canada). Lymphoprep™ should be protected from long exposure to light.

**1 – Isolation of Mononuclear Cells (MNCs) by Density Gradient Centrifugation**

1. Determine the total volume of the umbilical cord blood.
2. Aliquot a volume of density gradient medium equal to the volume of the umbilical cord blood into 50 mL conical centrifugation tubes (henceforth referred to as “50 mL tubes”). The maximum volume of density gradient medium in each 50 mL tube should not exceed 15 mL.
3. Dilute the umbilical cord blood using an equal volume of phosphate-buffered saline (PBS) or a 0.9% w/V solution of NaCl (saline).
4. Gently dispense the diluted umbilical cord blood onto the top of the density gradient medium. The volume of added blood should equal to double the volume of the density gradient medium.
5. Centrifuge the samples (400 g, 30 min, RT, with acceleration set to minimum and brakes disabled).
6. Transfer the MNCs forming the cloudy layer between the plasma and the density gradient medium into new 50 mL tubes. Ensure the maximum possible recovery of MNCs. Avoid pooling MNCs from different tubes.
7. Resuspend the MNCs in each 50 mL tube to 45 mL of total volume by adding PBS/saline and gently inverting the tubes multiple times.
8. Centrifuge the MNC suspensions (500 g, 5 min, RT).
9. Remove the supernatant by aspiration. Avoid decanting.
10. Resuspend the pelleted MNCs by adding 2 mL of PBS/saline and gently vortexing.
11. Add 20 mL of 1x Red Blood Cell Lysis Buffer (Cat.No. TNB-4300, Cytek Biosciences, USA) to each MNC suspension.
12. Gently invert the tubes multiple times to mix.
13. Incubate the MNCs in 1x Red Blood Cell Lysis Buffer (8 min, RT, protected from light).
14. Add magnetic-activated cell sorting (MACS) buffer (solution of 2 mM EDTA and 0.5% w/V bovine serum albumin in PBS, pH 7.2) into the MNC suspensions for a total suspension volume of 45 mL.
15. Gently invert the tubes multiple times to mix.
16. Centrifuge the MNC suspensions (300 g, 10 min, RT).
17. Remove the supernatant by aspiration. Avoid decanting.

* The MNCs can be frozen at this step for purification of CD34^+^ cells at a later timepoint. Once thawed, pooled and centrifuged, continue with the protocol from step 19.

1. If there are multiple tubes containing MNCs, resuspend each pellet in 1 mL of MACS buffer by pipetting and pool all of the MNC suspensions into a new 50 mL tube.

* From this point in the protocol onwards, work with cold solutions (4°C) and keep cells in a cold environment, unless specified otherwise.

1. Resuspend the MNCs in 40 mL of MACS buffer.
2. Filter the MNC suspension through a cell strainer with a pore size of 30-40 µm into a new 50 mL tube.
3. Determine the cell count.
4. Centrifuge the MNC suspension (300 g, 10 min, 4°C).
5. Remove the supernatant by aspiration. Avoid decanting.

**2 – Purification of CD34^+^ Human Hematopoietic Stem Cells**

1. Resuspend the MNCs in MACS buffer. The total volume of the MNC suspension should be 300 µL if there are 1 x 10^8^ MNCs or fewer. For higher cell counts, scale the suspension volume proportionally so there are 1 x 10^8^ MNCs per 300 µL of suspension. During the purification of hematopoietic stem cells, scale the volumes of used reagents in the same manner.

* The following CD34^+^ cell purification protocol has been adapted from that of the CD34 MicroBead Kit (human) (Cat. No. 130-046-703, Miltenyi Biotec, Germany).

1. For every 1 x 10^8^ cells, add 100 µL of human FcR blocking reagent into the MNC suspension.
2. For every 1 x 10^8^ cells, add 100 µL of CD34 microbeads into the MNC suspension.
3. Mix the MNC suspension by vortexing.
4. Incubate the MNC suspension on ice for 30 minutes, gently vortexing the suspension every 10 minutes.
5. For every 1 x 10^8^ cells, add 5 mL of MACS buffer to the MNC suspension.
6. Centrifuge the MNC suspension (300 g, 10 min, 4°C).
7. Remove as much of the supernatant as possible by aspiration. Avoid decanting.
8. For every 1 x 10^8^ cells, add 500 µL of MACS buffer to the MNC pellet.
9. Resuspend the pelleted MNCs by gently vortexing.
10. Move an aliquot of the MNC suspension containing 2 x 10^6^ cells into a separate tube for later flow cytometric analysis. Keep the aliquoted “pre-MACS” sample of MNCs cold.
11. Place an LS column into a MACS separator.
12. Rinse the LS column with 3 mL of MACS buffer and discard the flow-through.
13. Apply the MNC suspension onto the LS column and collect the flow-through fraction.
14. Wash the LS column with three 3 mL portions of MACS buffer and collect the flow-through fraction into the same tube as in step 37.
15. Move the LS column out of the magnetic field of the MACS separator and place it onto a 15 mL conical centrifugation tube.
16. Add 5 mL of MACS buffer onto the column and immediately force the added liquid through the LS column using the LS column’s plunger, collecting the eluate (the hematopoietic stem cell (HSC)-enriched fraction) into the 15 mL tube.
17. Perform steps 35-40 on the HSC-enriched fraction using a second LS column. Use the tube used in step 37 for the collection of unenriched flow-through fraction.
18. Determine the cell count in the flow-through fraction and the count and viability of the cells in the HSC-enriched fraction.
19. Move an aliquot of 1-2 x 10^6^ cells from the flow-through fraction and an aliquot of 1.5-2.5 x 10^4^ cells from the HSC-enriched fraction into separate tubes for flow cytometric analysis.
20. Keep the HSC-enriched fraction cold while determining HSC purity in cell aliquots via flow cytometry.

**3 – Determination of the Purity of Isolated CD34^+^ Human Hematopoietic Stem Cells**

1. Centrifuge the aliquots of pre-MACS, flow-through and HSC-enriched cells (450 g, 5 min, RT).
2. Remove as much supernatant as possible while minimizing cell loss.
3. Resuspend the pelleted cell aliquots. For every 5 x 10^6^ cells, rounded up, add 50 µL of PBS to the pellet.
4. Separate out an aliquot from the pre-MACS cell sample for use as an unstained control.
5. Stain the cell aliquots using an anti-human CD34 antibody (Cat.No. 130-098-140, Miltenyi Biotec, Germany).
6. Incubate antibody-stained cells for 10 minutes in a refrigerator (4°C).
7. Centrifuge the stained cell aliquots (450 g, 5 min, 4°C).
8. Aspirate supernatant.
9. Resuspend the cells in 250 µL of MACS buffer.
10. Acquire data on a flow cytometer.

**Supplementary Protocol 2: Preparation of Blood Samples for Spectral Flow Cytometry Analysis**

**1 – Thawing and Washing**

1. Thaw the cryovials containing the mouse blood samples fixed using Stable-Lyse2 (Cat.No STBLYSE2-250, Smart Tube, USA) and Stable-Store2 (Cat.No STBLSTORE2-1000, Smart Tube, USA) at 4°C.
2. While the samples are thawing:
   1. Label one 5 mL round-bottom tube for each sample.
   2. Prepare 2 mL of 0,25 mg/mL DNAse I (Cat.No. DN25, Sigma-Aldrich, USA) in Dulbecco’s phosphate-buffered saline containing Ca^2+^ and Mg^2+^ (Cat.No. D8662, Sigma-Aldrich, USA) per sample and aliquot 1 mL of the DNAse solution into each labeled 5 mL tube. Acclimate the DNAse solution to room temperature (RT).
   3. Acclimate CountBright Absolute Counting Beads (Cat.No. C36950, ThermoFisher Scientific, USA) to RT. Vortex the beads thoroughly. Into each labeled 5 mL tube containing DNAse solution, add 1 x 10^4^ counting beads for every 50 µL of blood (320 µL of fixed blood) constituting that sample.
3. One cryovial at a time:
   1. Pipet the contents of the cryovial gently and thoroughly to resuspend the cells.
   2. Transfer the contents of the cryovial into the corresponding 5 mL tube containing DNAse solution and counting beads.
   3. Add 1 mL of DNAse solution into the cryovial.
   4. Wash out the cryovial with the DNAse solution and transfer the washout to the corresponding 5 mL tube.
   5. Pipet gently and thoroughly to mix the contents of the 5 mL tube.
4. Incubate cells in DNAse solution for a minimum of 10 minutes.
5. Centrifuge the samples (800 g, 5 min, RT).
6. Remove supernatant by pipetting.
7. Add 1 mL of phosphate-buffered saline (PBS) to each sample.
8. Resuspend the cells by vortexing.
9. One sample at a time:
   1. Pipet the resuspended cells through the filter of a correspondingly labeled filter-capped 5 mL tube (Cat.No. 352235, Corning, USA).
   2. Add 1 mL of PBS to the original 5 mL tube.
   3. Wash the walls of the tube by vortexing.
   4. Transfer the washout through the filter of the corresponding 5 mL tube.
10. Centrifuge the samples (800 g, 5 min, RT).
11. Remove as much supernatant as possible by pipetting.

**2 – Staining**

1. Prepare FcR blocking buffer:
   1. 38 µL of base buffer (2% V/V fetal bovine serum in PBS) per sample.
   2. 10 µL of human FcR blocking agent (Cat.No. 130-059-901, Miltenyi Biotec, Germany) per sample.
   3. 2 µL of anti-mouse-CD16/CD32 monoclonal antibody (Cat.No. 16-0161-82, ThermoFisher Scientific, USA) per sample.
2. Add 50 µL of FcR blocking buffer to each cell pellet.
3. Resuspend the cells by gently vortexing.
4. Incubate cells in FcR blocking buffer for 10 minutes at RT.
5. Add 50 µL of antibody mix to each sample.
6. Mix by gently vortexing.
7. Incubate cells in the antibody mix for 30 minutes in a fridge (4°C).
8. Add 2 mL of base buffer to each sample.
9. Mix by vortexing.
10. Centrifuge the samples (800 g, 5 min, RT).
11. Remove supernatant by pipetting.
12. Repeat steps 19-22 to perform a second cell wash.
13. Resuspend the cells by gently vortexing.
14. One sample at a time:
    1. Measure the volume of the cell suspension using a pipette.
    2. Adjust the volume of the cell suspension to 200 µL using base buffer.
15. Acquire the samples on an ID7000 Spectral Cell Analyzer (LE-ID7000C, Sony Biotechnology, USA).

**Supplementary Protocol 3: Immunohistochemical Staining of Primary Mouse Tumor Sections**

**Day 1**

1. Bake the slides with the primary tumor sections at 60°C for at least 1h.
2. Cool the slides to room temperature (RT).
3. Deparaffinize and rehydrate the sections by incubation in xylene and a graded ethanol series:
   1. Xylene – pass 1 (10 min, RT).
   2. Xylene – pass 2 (3 min, RT).
   3. Ethanol (100%) – Pass 1 (3 min, RT).
   4. Ethanol (100%) – Pass 2 (3 min, RT).
   5. Ethanol (96%) – Pass 1 (3 min, RT).
   6. Ethanol (96%) – Pass 2 (3 min, RT).
   7. Ethanol (80%) (3 min, RT).
   8. Deionized water (diH_2_O) (5 min, RT).
4. Move the slides to a container of 1x Dako Target Retrieval Solution, pH 9 (Cat.No. S2367, Agilent, USA).
5. Perform heat-induced epitope retrieval. For example, by a 20-minute incubation in a microwave (Model JT366/WH, Whirlpool, USA) on a no-boil (“6^th^ sense”) setting.
6. Cool the slides in antigen retrieval solution on a benchtop for 10 minutes.
7. Further cool the slides to RT by placing the container of slides in antigen retrieval solution into a sink and pouring room-temperature diH_2_O into the container.
8. Dry the back of the slides and the area surrounding the tumor section using tissue paper.
9. Outline the tumor sections tightly using a PAP marker (Cat.No. Z627548, Sigma-Aldrich, USA).
10. Place the slides into hydration chambers containing moistened tissue paper.
11. To keep tumor sections hydrated while drying other slides, temporarily add diH_2_O onto tumor sections on dried slides.
12. Remove excess diH_2_O from the tumor sections by shaking the slides.
13. Add Dako Real™ Peroxidase Blocking Solution (Cat.No. S2023, Agilent, USA) onto the tumor sections.
14. Incubate for 10 minutes at RT.
15. Wash off the peroxidase blocking solution into a waste container by applying 1x Dako Wash Buffer (Cat.No. S3006, Agilent, USA) using a squeeze bottle. Temporarily leave a small amount of wash buffer on the tumor sections to keep them hydrated while washing other slides.
16. Remove excess wash buffer from the tumor sections by shaking the slides.
17. Add wash buffer onto the tumor sections.
18. Incubate for 5 minutes at RT.
19. Remove the wash buffer from the slides into a waste container.
20. For a second time, add wash buffer onto the tumor sections.
21. Incubate for 5 minutes at RT.
22. Wash off the wash buffer into a waste container by applying diH_2_O using a squeeze bottle. Sufficient wash buffer has been removed once the surface tension of the liquid on the slide allows the PAP marker outline to become clearly visible.
23. To keep tumor sections hydrated while washing other slides, temporarily add diH_2_O onto washed tumor sections.
24. Remove excess diH_2_O from the tumor sections by shaking the slides.
25. Add blocking solution (3% w/V bovine serum albumin (BSA) in phosphate-buffered saline (PBS)) onto the tumor sections.
26. Incubate for 45-60 minutes at room temperature.
27. Wash off the blocking solution into a waste container by applying wash buffer using a squeeze bottle. Temporarily leave a small amount of wash buffer on the tumor sections to keep them hydrated while washing other slides.
28. Remove excess wash buffer from the tumor sections by shaking the slides.
29. Add wash buffer onto the tumor sections.
30. Incubate for 5 minutes at RT.
31. Remove the wash buffer from the tumor sections into a waste container.
32. For a second time, add wash buffer onto the tumor sections.
33. Incubate for 5 minutes at RT.
34. Wash off the wash buffer into a waste container by applying diH_2_O using a squeeze bottle. Sufficient wash buffer has been removed once the surface tension of the liquid on the slide allows the PAP marker outline to become clearly visible.
35. To keep tumor sections hydrated while washing other slides, temporarily add diH_2_O onto washed tumor sections.
36. One slide at a time, remove as much excess diH_2_O as possible, avoiding damaging the tumor sections, then apply the appropriate primary antibody (diluted in 0.5% w/V BSA) to the tumor sections.
37. Incubate tumor sections in primary antibody overnight in a fridge (4°C).

**Day 2**

1. Retrieve the primary-antibody-stained tumor sections from the fridge.
2. Wash off the primary antibody solutions by tilting the slide over a waste container and applying wash buffer using a squeeze bottle.
3. Immediately place washed slides into a container of wash buffer.
4. Incubate on an orbital shaker for 5 minutes at RT.
5. Move the slides into a fresh container of wash buffer.
6. Incubate on an orbital shaker for 5 minutes at RT.
7. Wash off the wash buffer into a waste container by applying diH_2_O using a squeeze bottle. Sufficient wash buffer has been removed once the surface tension of the liquid on the slide allows the PAP marker outline to become clearly visible.
8. To keep tumor sections hydrated while washing other slides, temporarily add diH_2_O onto washed tumor sections.
9. Remove excess diH_2_O from the tumor sections by shaking the slides.
10. Apply the appropriate horseradish-peroxidase-conjugated secondary antibody (e.g. Dako EnVision+ System-HRP Labelled Polymer Anti-Mouse (Cat.No. K4001, Agilent, USA) or Dako EnVision+ System-HRP Labelled Polymer Anti-Rabbit (Cat.No. K4003, Agilent, USA)) to the tumor sections.
11. Incubate the tumor sections in secondary antibody for 30 minutes at RT.
12. Wash off the secondary antibody solutions by tilting the slide over a waste container and applying wash buffer using a squeeze bottle.
13. Immediately place washed slides into a container of wash buffer.
14. Incubate on an orbital shaker for 5 minutes at RT.
15. Move the slides into a fresh container of wash buffer.
16. Incubate on an orbital shaker for 5 minutes at RT.
17. Wash off the wash buffer into a waste container by applying diH_2_O using a squeeze bottle. Sufficient wash buffer has been removed once the surface tension of the liquid on the slide allows the PAP marker outline to become clearly visible.
18. To keep tumor sections hydrated while washing other slides, temporarily add diH_2_O onto washed tumor sections.
19. Remove excess diH_2_O from the tumor sections by shaking the slides.
20. Apply a solution of diaminobenzidine (Liquid DAB+, 2-component system (Cat.No. K3468, Agilent, USA)) to the tumor sections. Exercise caution while handling and disposing of diaminobenzidine due to its toxicity.
21. Incubate tumor sections in diaminobenzidine solution in the dark for 8 minutes at RT.
22. Thoroughly wash off the diaminobenzidine solution by tilting the slide over a toxic waste container and applying diH_2_O using a squeeze bottle.
23. To keep tumor sections hydrated while washing other slides, temporarily add diH_2_O onto washed tumor sections.
24. Remove excess diH_2_O from the tumor sections by shaking the slides.
25. Place the slides into a container of hematoxylin (Cat.No. S3301, Agilent, USA).
26. Incubate the tumor sections in hematoxylin for 10 minutes at RT.
27. Remove the slides from the hematoxylin and shake off the excess.
28. Place the slides into an empty container and wash the remaining hematoxylin off using several portions of warm tap water.
29. Place the slides into a container of diH_2_O.
30. Dehydrate the tumor sections and prepare them for mounting by dipping them 10 times into each container of a graded ethanol series and xylene:
    1. Ethanol (80%)
    2. Ethanol (96%) – Pass 1
    3. Ethanol (96%) – Pass 2
    4. Ethanol (100%) – Pass 1
    5. Ethanol (100%) – Pass 2
    6. Xylene – pass 1
    7. Xylene – pass 2
31. Mount the slides.
